# An MMP-degradable and conductive hydrogel to stabilize HIF-1α for recovering cardiac functions

**DOI:** 10.7150/thno.63481

**Published:** 2022-01-01

**Authors:** Xiaojuan Wei, Si Chen, Tian Xie, Hongchi Chen, Xin Jin, Jumin Yang, Shafaq Sahar, Huanlei Huang, Shuoji Zhu, Nanbo Liu, Changjiang Yu, Ping Zhu, Wei Wang, Wei Zhang

**Affiliations:** 1 Institute of Microsurgery on Extremities, Shanghai Jiao Tong University Affiliated Shanghai Sixth People's Hospital, Shanghai 200233, China.; 2 College of Chemical and Biological Engineering, Zhejiang University, Hangzhou 310027, China.; 3 School of Materials Science and Engineering, Tianjin Key Laboratory of Composite and Functional Materials, Tianjin University, Tianjin 300350, China.; 4 ZJU-Hangzhou Global Scientific and Technological Innovation Center, Hangzhou 311215, China.; 5 Department of Orthopedic Surgery, Shanghai Jiao Tong University Affiliated Shanghai Sixth People's Hospital, Shanghai 200233, China.; 6 Guangdong Cardiovascular Institute, Guangdong Provincial People's Hospital, Guangdong Academy of Medical Sciences, Guangzhou, Guangdong 510100, China.; 7 Department of Cardiovascular Surgery, Sun Yat-sen Memorial Hospital, Sun Yat-sen University, Guangzhou, Guangdong 510100, China.

**Keywords:** injectable hydrogel, myocardial infarction, matrix metalloproteinase, conductivity, hypoxia-inducible factor-1α

## Abstract

**Rationale:** Although a few injectable hydrogels have shown a reliable biosafety and a moderate promise in treating myocardial infarction (MI), the updated hydrogel systems with an on-demand biodegradation and multi-biofunctions to deliver therapeutic drug would achieve more prominent efficacy in the future applications. In this report, a conductive and injectable hydrogel crosslinked by matrix metalloproteinase-sensitive peptides (MMP-SP) was rationally constructed to stabilize hypoxia-inducible factor-1α (HIF-1α) to recover heart functions after MI.

**Methods:** Firstly, tetraaniline (TA) was incorporated into partially oxidized alginate (ALG-CHO) to endow the hydrogels with conductivity. The 1,4-dihydrophenonthrolin-4-one-3-carboxylic acid (DPCA) nanodrug was manufactured with high drug loading capacity and decorated with polymerized dopamine (PDA) to achieve a stable release of the drug. Both ALG-CHO and DPCA@PDA can be cross-linked by thiolated hyaluronic acid (HA-SH) and thiolated MMP-SP to construct a MMP-degradable and conductive hydrogel. After administration in the infarcted heart of rats, echocardiographic assessments, histological evaluation, and RT-PCR were used to evaluate therapeutic effects of hydrogels.

**Results:** The cell viability and the results of subcutaneous implantation verify a good cytocompatibility and biocompatibility of the resulting hydrogels. The hydrogel shows remarkable strength in decreasing the expression of inflammatory factors, maintaining a high level of HIF-1α to promote the vascularization, and promoting the expression of junctional protein connexin 43. Meanwhile, the multifunctional hydrogels greatly reduce the infarcted area (by 33.8%) and improve cardiac functions dramatically with ejection fraction (EF) and fractional shortening (FS) being increased by 31.3% and 19.0%, respectively.

**Conclusion:** The as-prepared hydrogels in this report achieve a favorable therapeutic effect, offering a promising therapeutic strategy for treating heart injury.

## Introduction

Myocardial infarction (MI) leads permanent damages to the heart muscle, which is one of the major causes of mortality worldwide 1, 2. The available treatments of MI include pharmaceutical therapy, medical device implants, and organ transplants, all of which have distinct limitations, such as high invasiveness, prolonged hospitalization time, re-plugging or restenosis, and scarcity of available donors 3, 4. Injectable biomaterials have emerged as a promising solution for cardiac tissue repair after MI. A large number of injectable hydrogels have been designed and delivered to the damaged area of a beating heart in a minimally invasive approach 5. IK-5001, an alginate hydrogel, demonstrates the therapeutic benefit on reducing the left ventricular (LV) enlargement in porcine and dog infarction models and also in clinical trials (NCT01226563) 6-8. Recently, a collagen hydrogel with autologous stem cells was employed in another clinical study (NCT02635464). A 12-month study showed no serious adverse effects 9. Although these hydrogels have confirmed the reliable biosafety in patients and are relatively promising in enhancing the repair of injured hearts, the updated hydrogel system with an on-demand biodegradation and multi-biofunctions to deliver therapeutic drug would achieve more prominent efficacy.

Cell infiltration and neo-tissue formation need to follow the pace of degradation of biomaterials, allowing the structural and functional integration of host tissue 10, 11. It was reported that the bio-inert, nondegradable polyethylene glycol (PEG) hydrogel could contribute to preserving LV wall thickness. Meanwhile, nondegradable hydrogel was insufficient to prevent post-MI remodeling, suggesting a limited therapy effect in rat MI model 12. To construct a cell-ingrowth matrix, the matrix metalloproteinase-sensitive peptides (MMP-SP) have been employed to construct the hydrogel networks with a rationally controlled degradation behavior which is triggered by over-expression of MMPs 13. Under pathophysiological conditions after MI, there is a persistence of over-expression and activity of MMPs that causes maladaptive changes in tissue architectures and functions 14-16. A few injectable hydrogels crosslinked by MMP-SP have recently been developed and applied post MI and the outcomes exhibited well-controllable deliverable therapeutics, contributing to a much better therapy effect 14, 16-18.

Another challenge for MI therapy is that the conduction of electrical impulse signals is interdicted because a fibrosis scar replaces normal myocardium in infarcted area 19-21. Conductive hydrogels may re-establish synchronous contraction and relaxation of a heart by facilitating the conduction of electrical impulse in infarcted area, which shows great benefits to the recovery of cardiac functions 22-24. In our previous work, the hydrogel modified by tetraaniline (TA) nanoparticles (NPs) showed a similar conductivity to that of native myocardium and resulted in a remarkable upregulation of the expression of junctional protein connexin 43 (Cx43) in the infarcted myocardium. Moreover, the incorporation of TA into the hydrogels does not exert a cardiotoxicity and arrhythmia on rat MI hearts 25, 26.

It is well known that an adult mammalian heart possesses a limited capability to regenerate itself. However, the hypoxia-inducible factor 1α (HIF-1α) has been recently explored to exert significant pro-regenerative effects by transcriptional regulation of various genes involved in metabolism, angiogenesis, and cell migration 27-30. Hence, the pharmacological prevention of HIF-1α degradation by prolyl hydroxylase (PHD) is emerging as a promising target in regenerative medicine 29, 31, 32. Recently, a small molecule drug, 1,4-dihydrophenonthrolin-4-one-3-carboxylic acid (DPCA) has been reported to be a potent inhibitor of PHDs and a stabilizer of HIF-1α both *in vitro* and *in vivo* 31, 33, 34. However, because of potential side effects associated with systemic delivery of hydroxylase inhibitors (including angiogenic and erythropoietic effects), it is desirable to develop new methods to achieve a target delivery of an efficacious and minimum amount of hydroxylase inhibitors to specific regions 35. On the other hand, the therapeutic applications of DPCA have been hindered by its intrinsic poor solubility and short half-life 33, 35. A mass of novel approaches based on nanoparticles has been developed as a smart delivery system to provide a perfect drug target 36. Among them, a simple and versatile particle modification strategy based on the famous dopamine polymerization has been well studied. Briefly, dopamine catechol can be conveniently and rapidly oxidized to quinone in a weak alkaline condition (∼pH 8.5) to shape a polymerized dopamine (PDA) layer on various and comprehensive surfaces with complexed morphologies. Due to the prominent advantages of simplicity and versatility, this methodology has widely been exploited to functionalize various types of substrates to stabilize the nanodrugs and achieve a stable and controllable drug release behavior 37-41. Additionally, the PDA layer will equip the modified nanoparticles with a reactive oxygen species (ROS)-scavenging capability 42 and can act as a crosslinker to react with thiol and amine groups 41, 43.

**Figure 1:**
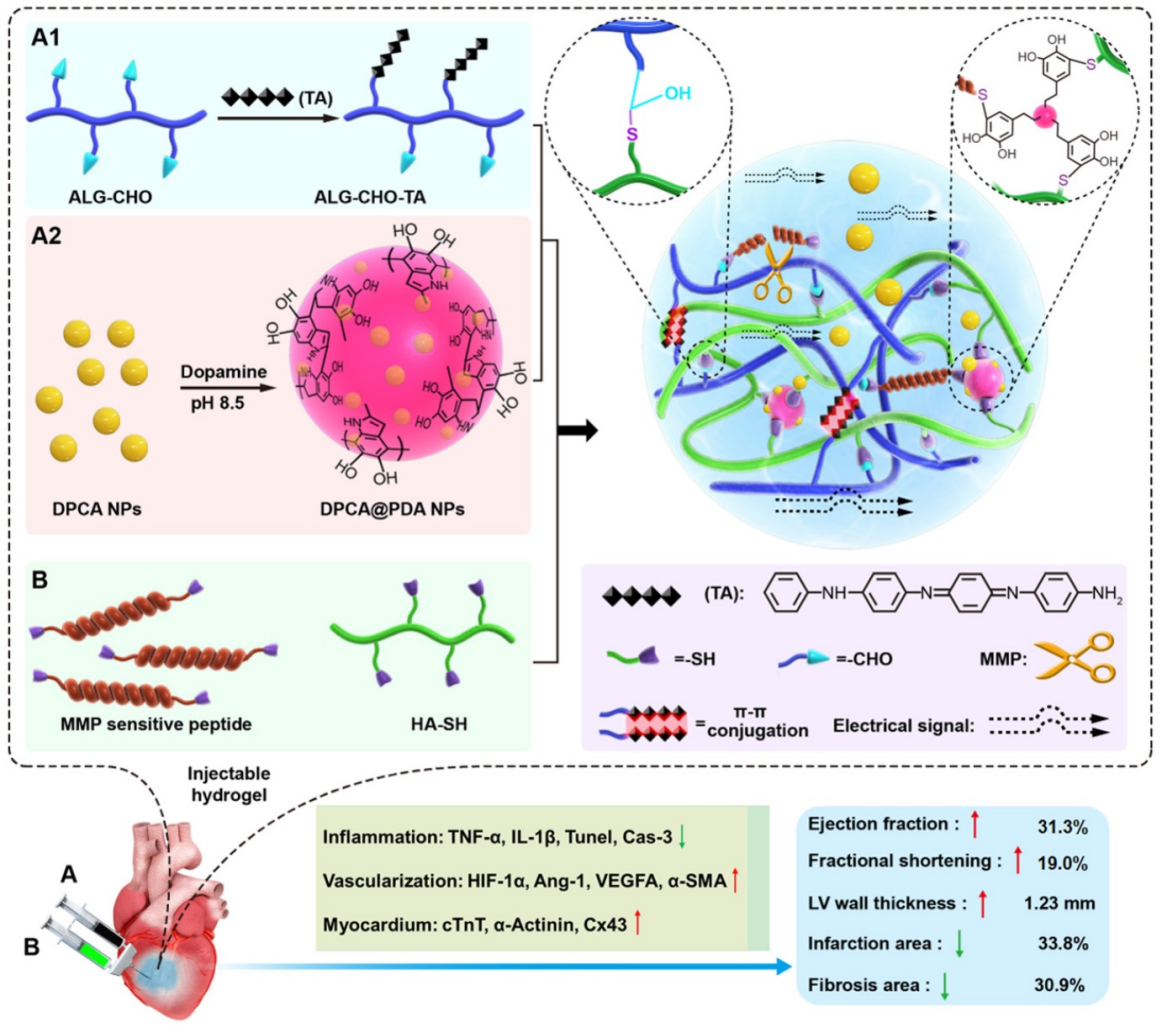
The schematic design of an MMP-degradable and conductive hydrogel to stabilize HIF-1α for recovering heart functions after MI. The hydrogel was fabricated based on functionalized HA and ALG. The conductive TA was incorporated into ALG-CHO to endow electric conductivity on the hydrogels. DPCA nanodrug was coated with PDA to achieve a high drug loading amount and maintain a stable releasing manner. Both ALG-CHO and DPCA@PDA are crosslinked by HA-SH and thiolated MMP-SP to construct the multifunctional hydrogels. The resulted hydrogels were administrated by intramyocardial injection into the infarcted rat hearts to evaluate therapy effects.

Calcium cross-linked alginate hydrogels are readily erodible in an uncontrollable manner and are associated with a high calcification risk 44-46. As illustrated in **Figure 1**, a multifunctional injectable hydrogel based on hyaluronic acid (HA) and ALG-CHO, which was cross-linked by MMP-SP to deliver DPCA, was rationally constructed to treat the infarcted rat heart. Firstly, TA was incorporated into ALG-CHO to endow the hydrogels with conductivity. The hydrophobic drug DPCA was fabricated into NPs by a reprecipitation method and then coated with PDA (DPCA@PDA) to stabilize the nanodrug. Both ALG-CHO and DPCA@PDA can be cross-linked by thiolated HA (HA-SH) and thiolated MMP-SP to construct the MMP-degradable and conductive hydrogel for restoring cardiac functions. After administration in the injured rat heart, the hydrogel could enhance the electrical conductivity and maintain a smart and sustained release of DPCA to stabilize HIF-1α in order to reverse the harmful microenvironment of infarcted zone consequently maintaining the heart functions in a better manner.

## Experimental Section

### Materials

Sodium alginate (ALG, low viscosity, 4-12 cP), sodium periodate (NaIO_4_), dopamine hydrochloride (DOPA), N-phenyl-p-phenylenediamine, and recombinant MMP-2 were purchased from Sigma-Aldrich. Thiol-modified hyaluronic acid (HA-SH, Mw = 300 kDa, degree of thiol substitution = 33%) was purchased from ESI BIO, USA. MMP-SP (Seq: CGPLG*GGRMSMPV*RDGSC where C is a thiol-containing cysteine and GGRMSMPV is the MMP-cleavable sequence.) was purchased from Bankpeptide Ltd., Hefei, China. DPCA was purchased from Santa Cruz Biotechnology Ltd. L929 Mouse fibroblast cell line (L929) and rat cardioblasts (H9C2) were purchased from National Biomedical Experimental Cell Bank, Beijing, China.

### Preparation and characterization of ALG-CHO-TA

ALG-CHO-TA was synthesized according to the previous reports 25, 26. Briefly, ALG (5 g) was dispersed in water (25 mL). The corresponding amount of NaIO_4_ aqueous solution according to the molar ratio of ALG (sugar moiety): NaIO_4_ was 2:1. This reaction was carried for 4 h in dark and under continuous stirring at 400 r/min. The reaction mixture was precipitated with 100% ethanol for 3 times to obtain ALG-CHO (a degree of oxidation = 42.8%, M_w_ = 4.2×10^5^ Da). In the next step, TA (0.93 g) was dispersed into 5mL N, N-dimethylformamide (DMF), and TA/DMF solution was added dropwise to ALG-CHO solution (1g ALG-CHO in 10 mL deionized water). The reaction was carried out for 4 h at 50 ^o^C under nitrogen atmosphere. The resulting liquid was precipitated with 100% ethanol for 3 times to acquire ALG-CHO-TA. ALG, ALG-CHO, TA, and ALG-CHO-TA were characterized by proton nuclear magnetic resonance (^1^H NMR) spectroscopy (Varian INOVA), ultraviolet-visible (UV-Vis) spectroscopy, and Fourier transform infrared (FTIR) spectrometry (PerkinElmer spectrum 100).

### Preparation and characterization of DPCA@PDA NPs

DPCA nanodrugs were fabricated by a typical reprecipitation method through a sudden change of solvent 37-41. Briefly, 400 μL of 25 mg/mL DPCA ethanol solution was added dropwise to 10 mL of water with a stirring rate of 900 r/min. The DPCA NPs with various sizes were prepared by adjusting the final concentration of DPCA. Subsequently, DOPA (9 mg) was dissolved in 5 mL buffer solution (pH 8.5), then an equal volume of DPCA NPs (1 mg/mL) aqueous dispersion was added, and the self-polymerization reaction was allowed to proceed under stirring for 12 h to obtain DPCA@PDA NPs. The resulting solution was centrifuged (15 min, 10000 r/min) and washed by ultrapure water for 3 times to obtain DPCA@PDA NPs. Dynamic light scattering (DLS, Malvern, Nano ZS) and transmission electron microscope (TEM, JEM-2100) were employed to assess the sizes and morphology of the resulting NPs.

### Preparation and characterization of the designed hydrogels

Precursor solution 'A' was obtained by mixing ALG-CHO-TA and DPCA@PDA solutions. Precursor solution 'B' was formed by mixing HA-SH with MMP-SP solution. Then, these two of solutions were then mixed with the volume ratio of A: B = 1: 2 to form the hydrogel. The final concentrations of various ingredients are shown in **Table S1**. The gelation time was measured by an inverted vial method.

Rheological measurements were performed by a rheometer (MCR 302). The time scan measurement was carried at the strain = 1% and frequency of 5 Hz. The amplitude sweep was performed with a frequency of 5 Hz. The shear thinning behavior was confirmed under the condition of 1% strain and frequency of 5 Hz. Anti-fatigue assessment was measured at a strain of 20% and a frequency of 5 Hz to simulate the beating rate of a normal rat heart.

The conductivities of the obtained hydrogels with different ratios and in PBS were measured via the four-point probe method (ST2253, China) as listed in **Table S2**.

A classic ROS clearance experiment was carried out to determine ROS scavenging capability. Briefly, a methanol solution of 1,1-diphenyl-2-picrylhydrazyl (DPPH) with UV absorbance at 516 nm was recorded and set as the initial state. 100 μL ALG-CHO, ALG-CHO-TA, DPCA, PDA, MMP-SP and HA-SH water solutions corresponding to the concentration in the hydrogel were prepared and added into 5 mL DPPH solution, and placed it in a shading environment at room temperature. 1 mL of the solution was taken to measure UV at regular time intervals, and then put it back until the absorbance at 516 nm disappeared. The ROS clearance ratio was calculated by the change ratio to the initial absorbance of DPPH solution.

### Drug release in vitro and in vivo

300 μL DPCA@PDA or ALG-CHO-TA/DPCA@PDA/MMP-SP/HA-SH hydrogel was placed in a dialysis bag (1000 Da). Then the dialysis bags were immersed into 50 mL PBS. The drug release behavior was carried out in a shake cultivation (300 r/min to simulate the beating rate of rat heart) under 37 ^o^C. To test the drug release profile with MMP, 5 U/mL MMP-2 was added in the solution. After a specific time interval, 3 mL solution was taken out and the same amount of fresh PBS was added. The resulting solution was assessed by UV-vis spectrum. The UV absorption of 261 nm was recorded to quantify the releasing rate of DPCA 29.

A florescent model drug was employed to determine the drug release behavior *in vivo*. Indocyanine green (ICG) is one of the FDA approved photosensitizers, which is safe and widely used in bioimaging and diagnose. In order to determine the drug releasing behavior *in vivo*, the fluorescent drug ICG instead of DPCA has been employed to prepare nanoparticles with the same procedure, and the resulted ICG nanoparticles was coated with PDA and crosslinked with the ALG-CHO-TA/MMP-SP/HA-SH hydrogels, and it was injected into the rat infarcted hearts (15 rats with ALG-CHO-TA/ICG@PDA/MMP-SP/HA-SH) to test the drug releasing behavior *in vivo*. The rat hearts were harvested 1, 3, 7, 14, and 21d after the operation, and the fluorescence intensity was measured by In Vivo Imaging System (PerkinElmer, IVIS Lumina II).

### Subcutaneous injection and establishment of MI model and injection of hydrogels

Male SD rats (220 ± 20 g) were supplied by Chinese Academy of Medical Science & Peking Union Medical College Institute of Biomedical Engineering. The protocols were in accordance with the Animal Care and Use Committee of Tianjin University and all procedures were performed on the 3R rules. The back hair of rats was removed after the rats were anesthetized. 15 rats were randomly divided into three groups and were subcutaneously injected with 200 μL hydrogels (n=5, III: ALG-CHO/HA-SH; IV: ALG-CHO-TA/HA-SH; V: ALG-CHO-TA/DPCA@PDA/HA-SH; VI: ALG-CHO-TA/DPCA@PDA/MMP-SP/HA-SH). In order to facilitate the description, the hydrogels used in subcutaneous injection and myocardial injection are grouped as the same. After 2, 4, and 7 d, the rats were over-anesthetized and sacrificed. Images of the skin tissue containing the hydrogel were taken to observe H&E staining.

As for the rat MI model, echocardiographic measurements were carried out before the surgery. The ejection fraction (EF) results were employed to screen rats. Those rats with an EF lower than 75% were gotten rid of the further research. The 114 SD rats were randomly divided into six groups (I: Sham; II: MI; III: ALG-CHO/HA-SH; IV: ALG-CHO-TA/HA-SH; V: ALG-CHO-TA/DPCA@PDA/HA-SH; VI: ALG-CHO-TA/DPCA@PDA/MMP-SP/HA-SH). The left artery was ligated after being anesthetized by a moderate flow of diethyl ether. The white infarcted area could be observed in the left heart. 100 μL hydrogel was injected by a 28-gauge needle in the MI area of two regions.

### Echocardiographic assessments

Echocardiography (Vevo 2100 Imaging System, Visual Sonics, Canada) was employed to access the left ventricular functions. The EF, fractional shortening (FS), left ventricular internal diameter (LVID), interventricular septum thickness (IVS), and left ventricular posterior wall thickness (LVPW) were calculated according to the averages of five consecutive cardiac cycles.

### Histological evaluation

The rats were euthanized at 3, 7, 14, and 28 d post implantation. All the hearts were placed in cardioplegic solution before fixation to ensure that all hearts are in a relaxed state. The hearts were dehydrated and cut into sections for Masson staining, H&E staining, Sirius red staining, and triphenyl tetrazolium chloride (TTC) staining. TTC staining sections were recorded by a digital camera. Masson staining, H&E staining, and Sirius red staining sections were observed by a fluorescence microscope (CKX41, Olympus, Japan). The Image J software was employed to calculate the ratio of infarction, fibrosis, and left ventricular wall thickness according to Masson staining sections.

### Immunofluorescent staining

The paraffin sections were deparaffinized and rehydrated with gradient alcohol. After rinsing with 0.01 mol/L PBS for 3 times, the slides were blocked by 10 % serum in a humid box at 37 ^o^C for 30 min. The primary antibody (**Table S3**) was added and then incubated in a 37 ^o^C humid box for 1 h and washed with PBS. Then fluorescent rabbit secondary antibody (1:200, Invitrogen) was dropped onto the slide that was subsequently incubated in a humid box in the darkness for 1 h. DAPI was added dropwise and the sections were incubated for 5 min in the dark. Finally, the slides were mounted with glycerol and the resulted slides were immediately observed and photographed by a fluorescence microscope (CKX41, Olympus, Japan).

### RT-PCR

The removed rat hearts were immediately frozen in liquid nitrogen. The quantitative real-time RT-PCR was carried out as previously reported 25, 26, 40. Briefly, the total RNA was extracted in strict accordance with the HiPure Plant RNA Mini Kit (R4151-03, MAGEN). A UV spectrophotometer was used to determine the RNA concentration. The total RNA (1 μg) was reversely transcribed to cDNA according to protocol. After the reaction was completed, the cDNA was diluted five times with sterile deionized water and stored at -20 °C. mRNA level of interleukin-1β (IL-1β), tumor necrosis factor (TNF-α), HIF-1α, Angiopoietin-1(ANG-1), α-Actinin, and cardiac troponin (cTnT) was detected (primer sequences in **Table S4**).

### Statistical analysis

The data is shown as means ± standard deviations. The statistical analysis is accessed by analysis of variance (ANOVA). P values < 0.05 were considered as significant (* indicates the p < 0.05 and ** indicates the p < 0.01).

## Results and Discussion

### Characterization of ALG-CHO-TA

TA was chosen and employed as a conductive constituent to graft ALG-CHO by a Shift-base reaction between amine and CHO groups (**Figure S1**). The chemical structure of ALG, ALG-CHO, TA, and ALG-CHO-TA was assessed by ^1^H-NMR. As indicated in **Figure S2**, the peak at 8.3 ppm attribute to the aldehyde group and the peak at 5.6 ppm attributes to the hemiacetal group, confirming ALG-CHO was successfully synthesized. The proton peaks appear at 6.5-7.5 ppm, representing the proton on benzene ring (**Figure S2**). As shown in **Figure S3**, the peaks of 3382 and 3030 cm^-1^ represent the stretching vibration of N-H and Ar-H, suggesting the successful synthesis of ALG-CHO-TA. A UV-vis standard curve of TA was fabricated to determine the amount of TA in ALG-CHO-TA polymer chain. It is calculated that the solid content of TA in ALG-CHO-TA is 8.9 wt% and the grafting efficiency of TA is 33.8%.

### Properties of DPCA and DPCA@PDA NPs

**Figure 2:**
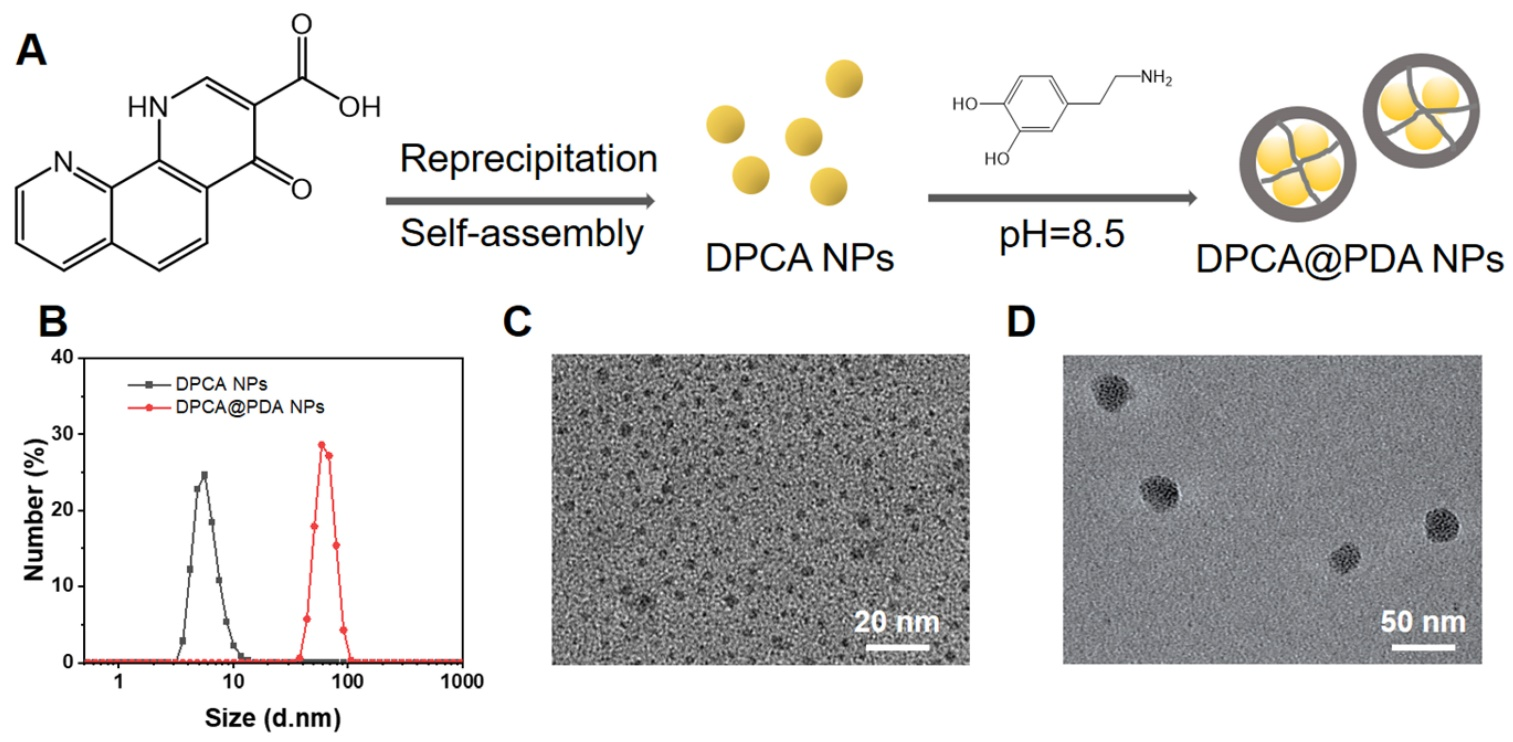
The synthesis and characterization of DPCA@PDA NPs. (A) The synthesis process of DPCA@PDA. (B) The size distribution of DPCA and DPCA@PDA NPs assessed by DLS. TEM images of DPCA NPs (C) and DPCA@PDA NPs (D).

The DPCA@PDA NPs were fabricated as the procedures shown in **Figure 2A**. As demonstrated in **Figure S4**, the diameters of DPCA NPs are 6.4, 40.7, and 81.2 nm when the final concentrations of DPCA are 1, 2, and 5 mg/mL, indicating a higher concentration will lead to large sized NPs. Because a small size is more suitable for PDA coating, the low concentration (1 mg/mL) of DPCA solution was selected for further experiments. After coating with PDA, the diameter of NPs increased from 6.4 to 74.8 nm (**Figure 2B**). The uniformly dispersed DPCA NPs are obtained in the TEM image (**Figure 2C**). As indicated in **Figure 2D**, several DPCA nanoparticles are encapsulated by the PDA crosslinked network, which looks like a pomegranate fruit. The sizes of DPCA and DPCA@PDA NPs are about 3.7 and 32.8 nm according to TEM images.

### Properties of the hydrogels

A typical thiol-aldehyde Michael addition can take place between aldehyde group of ALG-CHO and thiol groups of HA-SH to form the hydrogel network 47. In addition, DPCA@PDA can react with a thiolated polymer chain through a thiol-benzoquinone Michael Addition reaction. Meanwhile, thiol end capped MMP-SP can participate in the crosslinking reaction to entitle a MMP induced degradability to the hydrogels.

**Figure 3:**
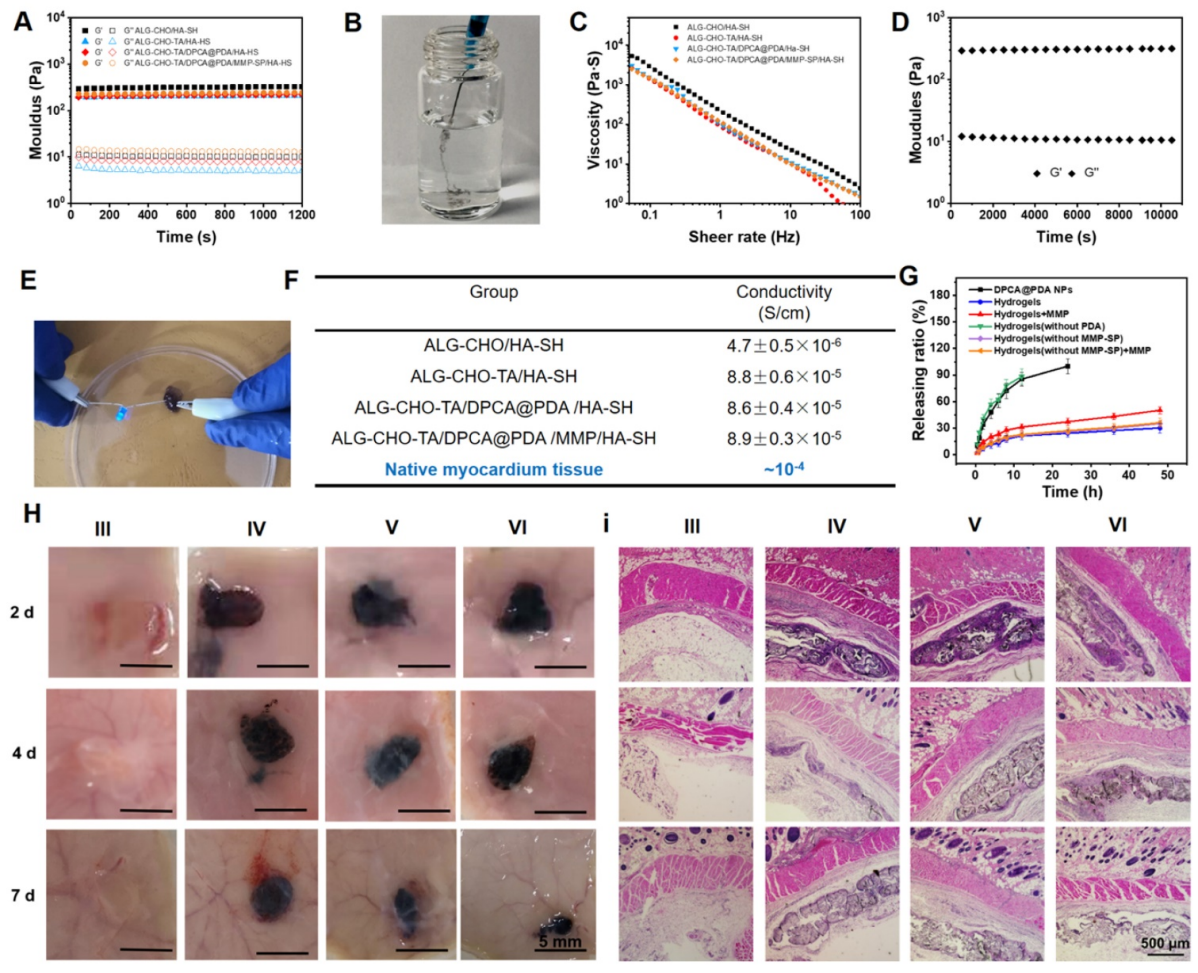
Properties of the as-prepared ALG-CHO-TA/DPCA@PDA/MMP-SP/HA-SH hydrogels. (A) Rheological analysis based on a time-sweep model. (B) The macro picture to demonstrate the injectable capability of the hydrogels. (C) The shear-thinning behavior and (D) the fatigue resistance of ALG-CHO-TA/DPCA@PDA/MMP-SP/HA-SH hydrogel. (E) The macro picture indicates that the conductive hydrogel can act as a wire to light up LED. (F) The conductivity of the hydrogels. (G) The DPCA releasing behavior in vitro. (H) The macro pictures illustrate the subcutaneous injection of hydrogels within 7 d. (I) H&E staining of the tissue around the hydrogels (black zone in the images) after 7-day subcutaneous injection. (III: ALG-CHO/HA-SH; IV: ALG-CHO-TA/HA-SH; V: ALG-CHO-TA/DPCA@PDA/HA-SH; VI: ALG-CHO-TA/DPCA@PDA/MMP-SP/HA-SH.)

A parallel-plate rheometer was employed to determine rheological properties of the resulting hydrogels. As shown in the time-sweep curve (**Figure 3A**), G' is larger than G'' during the whole time period, revealing a crosslinked network before the samples were been placed on the rheometer plate. A typical G'/G'' crossover can hardly be observed due to the rapid sol-gel transition (**Table S1**). The hydrogels with various components possess a similar and soft mechanical property (200-300 Pa). The gelation time can be adjusted by the concentration of HA-SH. As shown in **Table S1**, the gelation occurs within 30 s when the concentration of HA-SH is 1.33%. The addition of DPCA@PDA and MMP-SP exerts fewer effects on gelation time and shows a slight increase in the mechanical property. The injectable capability can be clearly visualized in **Figure 3B,** suggesting that the hydrogel can be injected into water and maintain a stable shape.

A typical shear thinning behavior can be observed in **Figure 3C.** The viscosity of the hydrogel goes down from ~3000 to ~1 Pa•s as the shear rate rises up. Meanwhile, maintaining a stable rheological property is important for hydrogels used in a continuous beating heart. The G' and G'' of the hydrogels remain stable at the strain range of 0.1%-100% and the frequency from 0.5 to 100 rad/s (**Figure S5-6**). To verify a long-time stability of the hydrogel in a beating heart environment, the resulting hydrogel maintains stable after a 12000-time continuous shear (**Figure 3D**).

After MI, the injury induces fibroblast proliferation to secrete extracellular matrix forming the scar tissue, then a blockage of the transmission of electrical signals occurs 21, 48. The conductive TA is reported to effectively propagate the electrical signals in the infarcted area 25, 26. As revealed in the UV-vis spectrum of TA and ALG-CHO (**Figure S7**), there is a single peak at 325 nm of TA and ALG-CHO-TA, attributing to π-π* transition of benzene ring. However, the noticeable peak at 422 nm confirms the existence of eigenstate TA after HCl-doping, which ensures the conductivity of TA. The ALG-CHO-TA/HA-SH hydrogel can light up LED as a wire (**Figure 3E**). As shown in **Figure 3F**, the addition of TA greatly improves the conductivity of hydrogels. The hydrogel (ALG-CHO/HA-SH) without TA possesses a conduction of 4.7×10^-7^ S/cm. The conductivity of hydrogel with TA is ~8.8×10^-5^ S/cm, which is consistent with the conductivity of native myocardium (5×10^-5^-1.6×10^-3^ S/cm) 49. Meanwhile, the TA containing hydrogels were immersed in PBS to test the conductivity. As shown in **Table S2**, the hydrogel is immersed in PBS for 24 h, the conductivity increases slightly, which might attribute to the absorption of ions in PBS to increase ion conductivity.

It is well reported that reactive oxygen species (ROS) are key signaling molecules that play an important role in the progression of inflammatory disorders. An enhanced ROS generation by polymorphonuclear neutrophils (PMNs) at the site of inflammation causes cell dysfunction and tissue injury. A classic ROS clearance experiment was carried out to determine the ROS scavenging capability of the designed hydrogels. Briefly, ALG-CHO, ALG-CHO-TA, DPCA, PDA, MMP-SP and HA-SH water solutions corresponding to the concentration in the hydrogel were prepared and added into the ethanol solution of DPPH. The absorbance at 516 nm of the mixed solutions were used to determine the ROS removal efficiency of six components. As shown in **Figure S8**, all the six components illustrate a different capability to scavenge ROS. PDA possesses the highest ROS clearance efficiency. 83.2% of ROS has been cleared at 7 min, and the clearance rate of ROS can reach 96.7% after 48 h. In addition, compared with PDA and ALG-CHO-TA, which rapidly clears ROS in the first 2 h and then gradually slows down, the ROS clearance rate of HA-SH maintains stable in the first 10 h, and the final ROS clearance efficiency is even higher than that of ALG-CHO-TA. This may be due to the fact that the concentration of HA-SH in the hydrogel is much higher than that of other components, which can gradually scavenge free radicals in DPPH. The experimental results show that each component of the hydrogel has a certain ROS scavenging effect, indicating a ROS clearance ability of the hydrogel.

As illustrated in **Figure 3g**, DPCA in DPCA@PDA NPs can quickly diffuse to PBS solution within 24 h. The DPCA drug exhibits a very fast release rate (90% drug released within 12 h) when it is loaded in the hydrogel without a PDA layer. On the other hand, the drug releasing maintains a stable and much slower speed after being loaded in the hydrogels (30% drug released after 48 h). The drug release behavior can be sped up by adding MMP in the solution (50% drug released after 48 h). These outcomes indicate that DPCA can be triggered to a faster release in an infarcted heart due to significant amount of over-expressing MMPs. The releasing rate of DPCA in the hydrogel without the addition of MMP-SP was only slightly increased, which may be attributed to a higher crosslinking density due to the addition of MMP-SP in the hydrogel. Regardless of whether MMP is added or not in the media, the release rate of DPCA in the hydrogel without MMP-SP is basically the same. These results prove that MMP-SP confers a MMP sensitivity on the hydrogels and both the hydrogel and PDA layer will enhance the controllable release capability of DCPA drug. The experiments of drug release were stopped due to the drug release behavior approaching to a linear manner after the first 24 h and maintaining a stable tendency in the following 24 h. As indicated in **Figure 3G**, 12 % drug is released within the next 24 h. It is established that DPCA drug in the hydrogel could be kept for 6 d. However, the microenvironment of infarcted myocardium is sharply different from that *in vitro*.

### Cytocompatibility and the biocompatibility of the hydrogels

In order to verify cell biocompatibility, the extract medium of various hydrogels was employed as the medium to culture H9C2 and L929. As exhibited in **Figure S9**, the cell viability is higher than 90% within 48 h, indicating a negligible cytotoxicity of the resulted hydrogels. Meanwhile, the cytocompatibility was characterized by the cells seeding on the top of hydrogels. As indicated in **Figure S10**, all the hydrogel samples exhibit a higher cell viability than 90%, suggesting an excellent cytocompatibility. Meanwhile, a typical live/dead viability analysis have been carried out. As displayed in **Figure S11**, the live cells were stained green by Calcein-AM and the dead cells were stained red by propidium iodide (PI), respectively. A majority of H9C2 cells were stained green and few cells were stained red, indicating an excellent outstanding biocompatibility.

To evaluate the anti-apoptosis effects of DPCA, a doxorubicin (Dox) induced cell apoptosis was employed. The DPCA concentration was fixed at 6.8 μM, equally as it released form hydrogels in 24 h under a simulated pathological environment. The cell viability was evaluated by CCK-8 method. As shown in **Figure S12**, DPCA can significantly inhibit CM apoptosis. The regulating effects of HIF-1α expression of H9C2 cultured with DPCA-loading hydrogels (DPCA@PDA gel) were verified by both PCR and western-blotting (WB). The PDA gel without DPCA was used as a control to exclude the potential interference effects by the other components of the hydrogels. As shown in **Figure S12**, both PCR and WB results show that not only the gene expression of HIF-1α is promoted, but also the protein level of HIF-1α is increased by DPCA-loaded hydrogel, indicating its ability of activating and stabilizing HIF-1α pathway by DPCA@PDA hydrogel.

Subcutaneous injection was carried out to estimate the biocompatibility and degradable behavior of the designed hydrogels. As displayed in **Figure 3H**, the hydrogels do not cause severe inflammatory responses within 7 d. In addition, all the hydrogels degrade gradually *in vivo* while the hydrogel with MMP-SP (Group VI) gains a much faster degradable rate than those hydrogels without MMP-SP. At 7 d post-injection, it was clearly found that more blood vessels appear around ALG-CHO-TA/DPCA@PDA/HA-SH and ALG-CHO-TA/DPCA@PDA/MMP-SP/HA-SH hydrogel, attributing to the angiogenesis of DPCA. The H&E staining results (**Figure 3I**) show that there are no visible inflammatory responses in subcutaneous tissue, further confirming a good biocompatibility of hydrogels. Quantitative results on vascular density were shown in **Figure S13**. Comparing with group III and IV, the vascular densities of group V and VI were higher, which could confirm the effect of DPCA in promoting vascular regeneration.

### Evaluation of biosafety and heart functions after the hydrogel injection

As shown in **Figure S14**, the fluorescent intensity of ICG in the hearts is rapidly weakened within the first 3 d. As calculated by Image J, the drug releasing rate exceeds 95% after 3 d. As demonstrated in **Figure S15**, there are no apparent lesions or abnormalities and significant inflammatory responses in the main organs (heart, liver, spleen, lung, and kidney). The morphology of main organs keeps the same with that of normal rats. The experimental results reveal that the injection of hydrogels with DCPA and TA nanoparticles would not cause a significant inflammation and other seriously negative effects on the main organs of rats.

**Figure 4:**
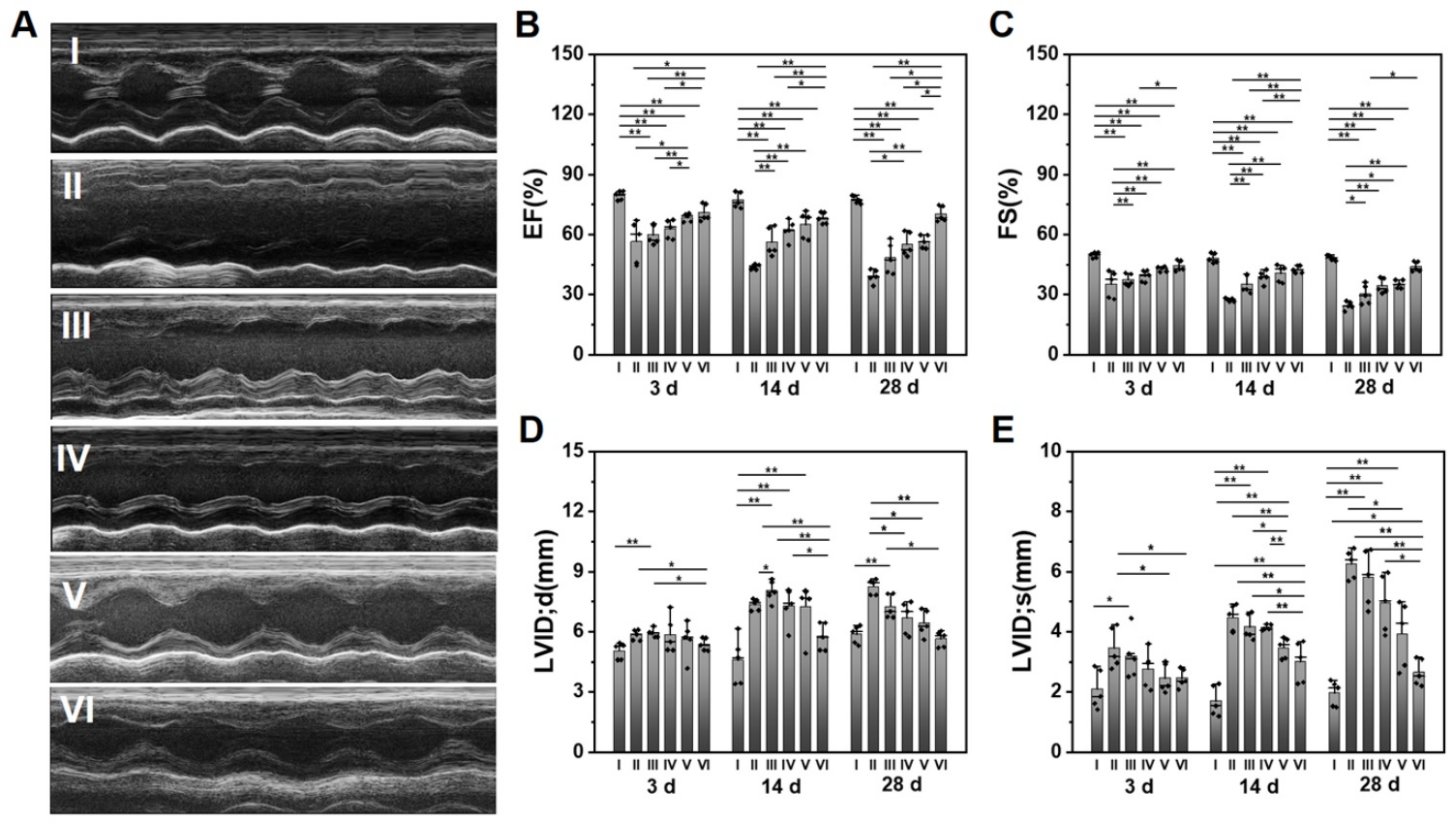
Evaluation of the cardiac functions after various treatments at different time points. (A) The representative echocardiographic images for various groups at 28 d. The statistical values of (B) EF, (C) FS, (D) LVID; d, and (E) LVID; s. (I: Sham; II: MI; III: ALG-CHO/HA-SH; IV: ALG-CHO-TA/HA-SH; V: ALG-CHO-TA/DPCA@PDA/HA-SH; VI: ALG-CHO-TA/DPCA@PDA/MMP-SP/HA-SH.)

The survival rate of rats in each group within 28 d can be observed in **Table S5**. 16% MI rats die during the experiment. Heart functions were measured by an echocardiography at 3, 14, and 28 d after surgery. The typical echocardiographic images at 28 d are shown in **Figure 4A**. The statistical data on EF, FS, and LVID is displayed in **Figure 4B-E**, while the data on IVS and LVPW is shown in **Figure S16**. The EF and FS of infarcted hearts (Group II) continue to decline over time, while it is effectively suppressed by the hydrogel injection. The ALG-CHO-TA/DPCA@PDA/MMP-SP/HA-SH hydrogel achieves the best effects (EF=70.2 ± 3.9%, FS=43.9 ± 2.4%). As depicted in **Figure 4D-E**, the LV diameter of MI rats (Group II) is much longer than that of normal heart (Group I), which is shortened by hydrogels (Group III to VI). Data on IVS and LVPW demonstrates the similar trend with that of EF and FS, further certificating the therapeutic effects of hydrogels on heart functions. All these data verify that the injection of the conductive and MMP-degradable hydrogel performs excellent benefits on restoring heart functions. As shown in **Figure 4**, the hydrogels exhibit an instant effect on the myocardial functions witnessed by the higher EF and FS than that of infarcted hearts. These effects might be attributed to the soft and biocompatible hydrogels in the infarcted zone to capture ROS produced by the myocardial ischemia and to reduce the death of cardiomyocytes (CMs). With the time extension after MI, the myocardial functions further degenerate, which can be verified by a decrease in EF and FS (**Figure 4B-C**). The previous studies have demonstrated that DCPA possesses an ability of tissue regeneration 29, 33. In this study, DCPA can maintain a high level of HIF-1α to promote the vascularization and preserving heart functions for 28 d.

### Histological evaluation of heart tissue

**Figure 5:**
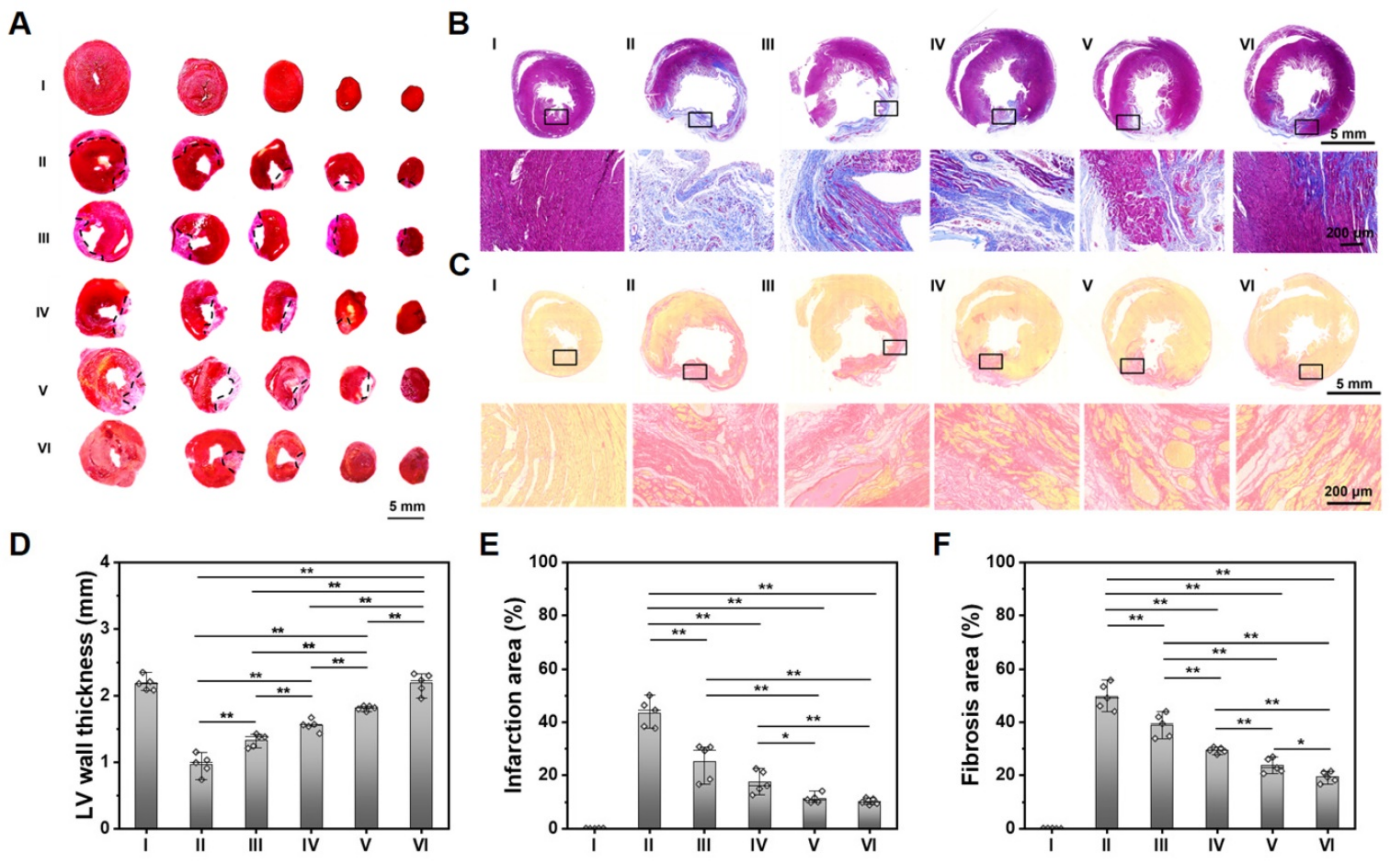
Histological analysis of the cardiac tissue at 28 d. (A) TTC staining. (B) Masson staining. (C) Sirius red staining. Statistical data on LV wall thickness (D), the infarcted area (E), and the fibrosis area (F). (I: Sham; II: MI; III: ALG-CHO/HA-SH; IV: ALG-CHO-TA/HA-SH; V: ALG-CHO-TA/DPCA@PDA/HA-SH; VI: ALG-CHO-TA/DPCA@PDA/MMP-SP/HA-SH.)

As shown in **Figure 5A-C**, compared with the obvious infarcted area and thin LV wall of a non-treated MI heart, those hearts treated with ALG-CHO-TA/DPCA@PDA/MMP-SP/HA-SH hydrogel hold the slightest collagen deposition and the thickest LV wall thickness. The statistical results are shown in **Figure 5D-F**, compared with the normal hearts (Group I), the LV wall thickness in the center of infarcted zone decreases to 0.97 mm, and the infarcted area and fibrosis area reach to 43.9% and 50.1%. After being treated by ALG-CHO-TA/DPCA@PDA/MMP-SP/HA-SH hydrogels (Group VI), the LV wall thickness in the center of infarcted zone can be maintained to 2.20 mm, and the infarcted area and fibrosis area decrease by 33.8% and 30.9%, respectively.

To further illustrate the myocardial structure of hearts treated with various hydrogels, a much-detailed information is depicted in **Figure 5** and **S17**. The myocardial tissue of MI heart (Group II) is disordered after MI, which is relieved over time. There is still inflammatory cell infiltration in a non-treated MI heart (Group II) at 28 d. For the heart treated with the ALG-CHO-TA/DPCA@PDA/MMP-SP/HA-SH hydrogel, the inflammatory microenvironment is significantly alleviated, and the myocardial tissue in the infarcted zone is partly arranged.

### Immunofluorescent analysis and RT-PCR

**Figure 6:**
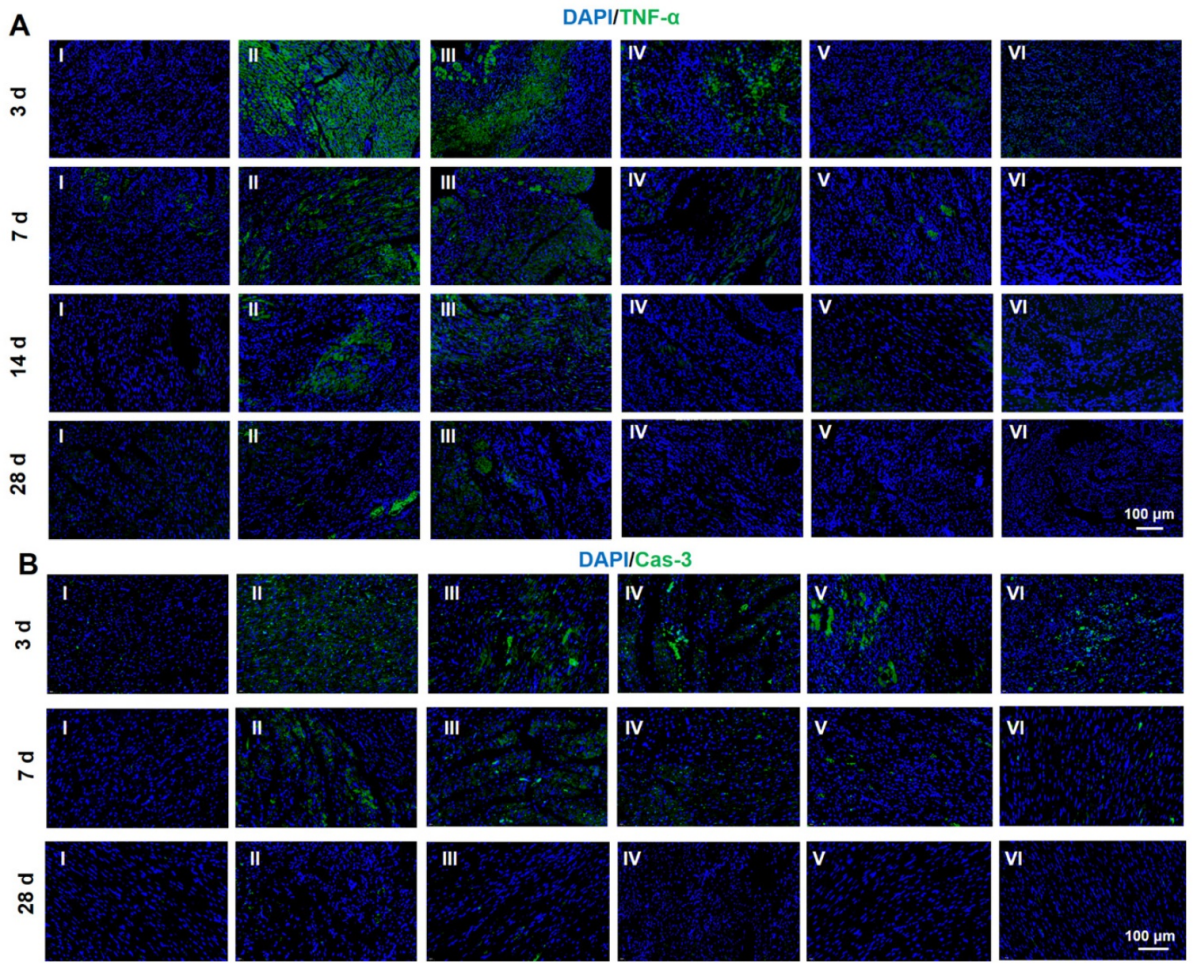
Protein expression level of TNF-α (A) and Cas-3 (B) determined by immunofluorescent staining at 28 d. (I: Sham; II: MI; III: ALG-CHO/HA-SH; IV: ALG-CHO-TA/HA-SH; V: ALG-CHO-TA/DPCA@PDA/HA-SH; VI: ALG-CHO-TA/DPCA@PDA/MMP-SP/HA-SH.)

It is significant for the treatment of MI to fight the inflammation caused by the myocardial necrosis and promote the regeneration of vessels 50. Thus, inflammatory factors and angiogenesis-related factors are frequently used as the indexing factors 51-54. Both immunofluorescent analysis and RT-PCR were employed to evaluate the expression of related factors. In this research, TNF-α and caspase-3 (Cas-3) were selected to index inflammatory factors and cell apoptosis. As indicated in **Figure 6A** and** S18**, the immunofluorescent staining of TNF-α is clearly observed after MI at 3 d. The amount of TNF-α is declined by the hydrogel injection. As the time span increases to weeks, the amount of TNF-α is sharply reduced due to the host adaptation. However, the hydrogels exert a strong power on the down-regulation of TNF-α. The same tendency can be found in the expression of Cas-3, suggesting an obvious inhibition in the cell apoptosis. In addition, the staining of Tunel was conducted to determine the apoptosis of CMs. As displayed in **Figure S19-20**, the hydrogels loaded with DPCA@PDA and MMP-SP show the best protection on CMs against the severe microenvironment in the infarcted heart. All these data confirm that the multifunctional hydrogel has distinct anti-inflammatory effects to reverse the undesirable microenvironments of infarcted heart.

**Figure 7:**
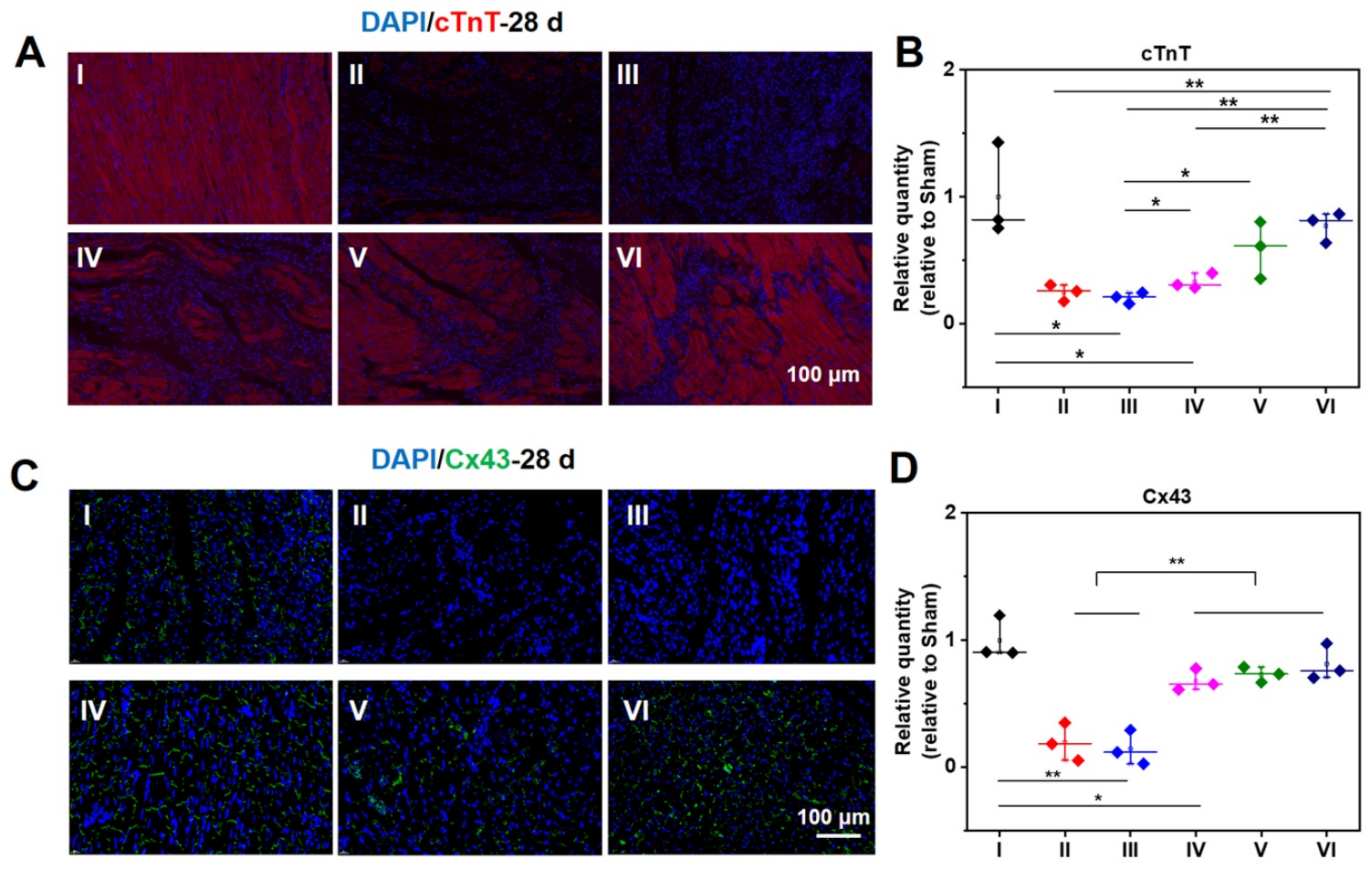
Immunofluorescent staining of cTnT (A, B) and Cx43 (C, D) at 28 d. B, D are the statistical data quantified and normalized to the number of nuclei. (I: Sham; II: MI; III: ALG-CHO/HA-SH; IV: ALG-CHO-TA/HA-SH; V: ALG-CHO-TA/DPCA@PDA/HA-SH; VI: ALG-CHO-TA/DPCA@PDA/MMP-SP/HA-SH.)

Meanwhile, cTnT and Cx43 were selected to ascertain the functions of CMs and cell to cell communications in the infarcted zone. In addition to the acutely affected area, CMs in the adjacent zones of survival are also prone to death after MI. As a result, the expression of cTnT will significantly reduce in the center and adjacent zone of infarcted hearts. As demonstrated in **Figure 7A**, compared with a normal heart, the expression of cTnT of infarcted myocardium at 28 d is distinctly descended. The expression of cTnT in the infarcted hearts treated by hydrogels elevates to a large degree. For the injured hearts treated with the ALG-CHO-TA/DPCA@PDA/MMP-SP/HA-SH hydrogel, the expression of cTnT is significantly up-scaled, and CMs is orderly arranged. The amount of cTnT in Group VI shows a similar response as that of normal hearts (Group I) (**Figure 7A**). As displayed in the statistical data in **Figure 7B**, the increase in cTnT is attributed to a much better survival rate of those CMs in the infarcted area. The well biocompatible hydrogel system can inhibit the overexpression of inflammatory factors and improve the microenvironment of injured hearts, thereby effectively improving the function of partly injured CMs in the infarcted tissue.

As revealed in **Figure 7C-D**, the expression of Cx43 is sharply decreased after MI, suggesting a remarkable loss in cell to cell communications. Meanwhile, the expression of Cx43 in the conductive hydrogel treatment group (Group IV, V, and VI) is remarkably enhanced, suggesting an enhanced cell to cell communications and the electrical coupling in the infarcted zone.

**Figure 8:**
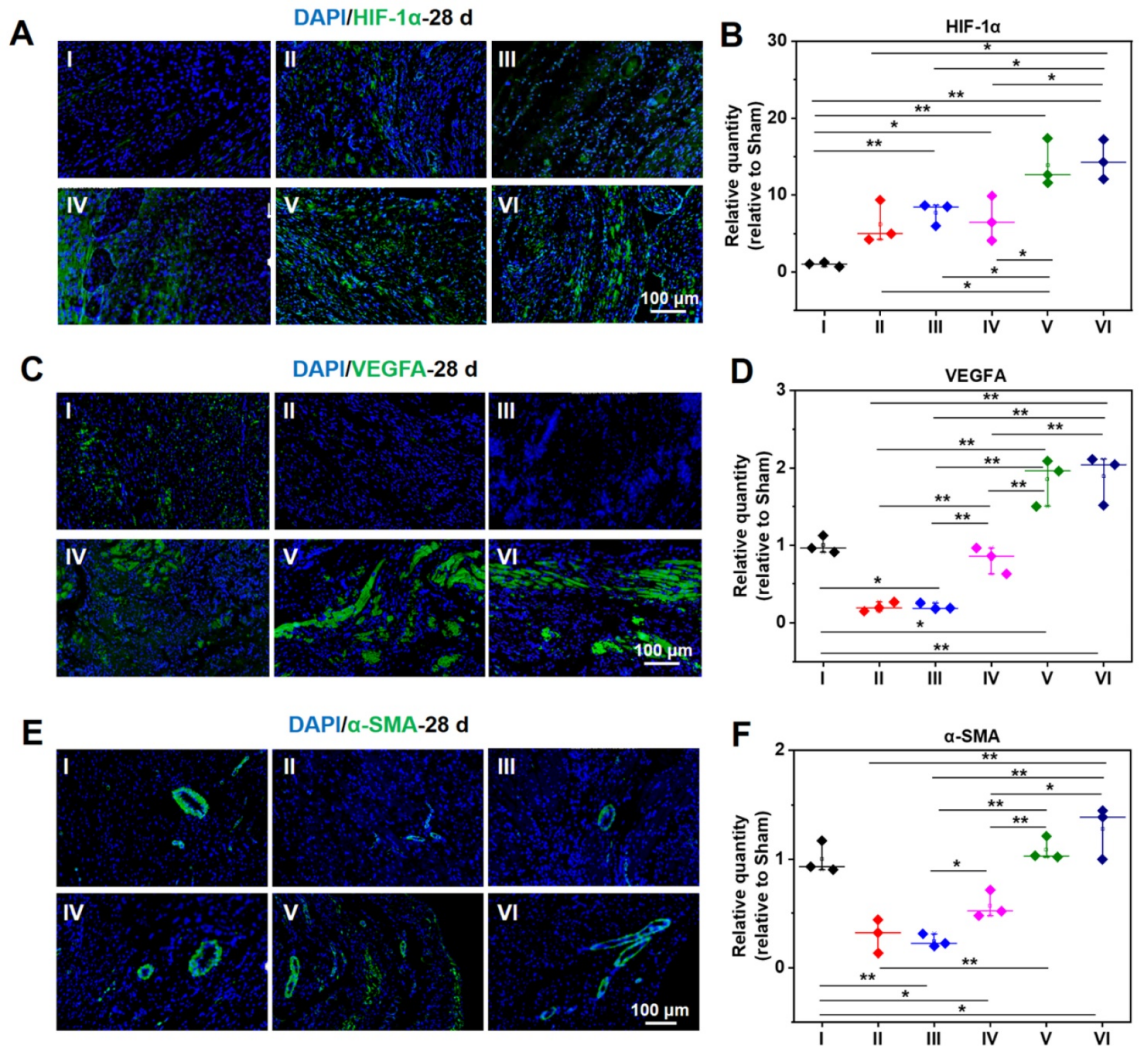
Protein expression level of HIF-1α (A, B), VEGFA (C, D), and α-SMA (E, F) at 28 d measured by immunofluorescent staining. B, D, and F are the statistical data quantified and normalized to the number of nuclei. (I: Sham; II: MI; III: ALG-CHO/HA-SH; IV: ALG-CHO-TA/HA-SH; V: ALG-CHO-TA/DPCA@PDA/HA-SH; VI: ALG-CHO-TA/DPCA@PDA/MMP-SP/HA-SH.)

As demonstrated in **Figure 8A-B**, the expression of HIF-1α in a normal heart is barely distinguishable, while the expression of HIF-1α in an infarcted heart is raised to a certain degree. The infarcted hearts treated by hydrogel loaded DCPA gain a much higher expression of HIF-1α. As shown in **Figure 8C-F**, the same tendency can be extended to the expression level of VEGFA and α-SMA, demonstrating a strong capability of angiogenesis.

**Figure 9:**
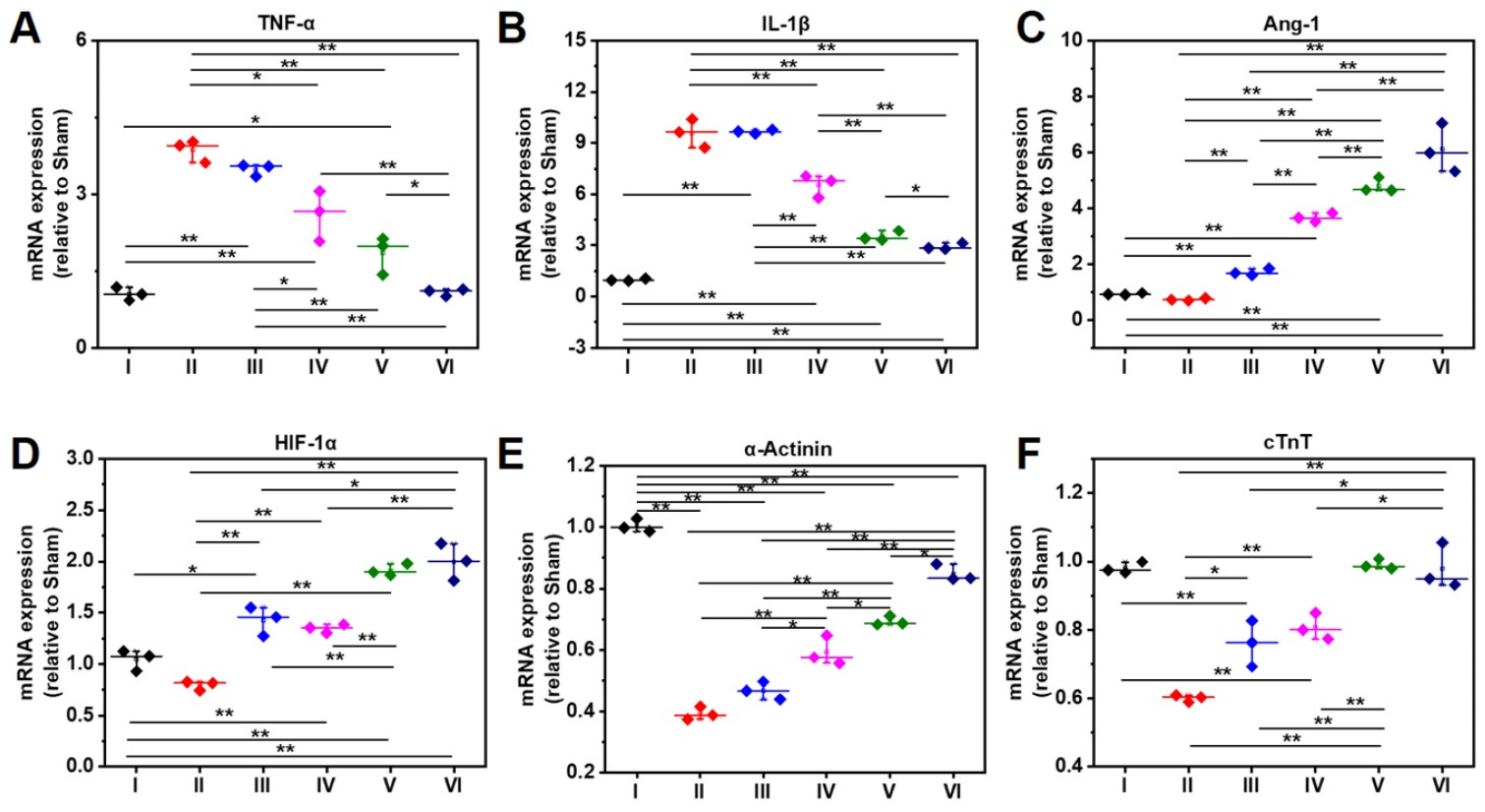
Gene expression level at 28 d determined by RT-PCR. A-F indicate TNF-α, IL-1β, Ang-1, HIF-1α, α-Actinin, and cTnT. (I: Sham; II: MI; III: ALG-CHO/HA-SH; IV: ALG-CHO-TA/HA-SH; V: ALG-CHO-TA/DPCA@PDA/HA-SH; VI: ALG-CHO-TA/DPCA@PDA/MMP-SP/HA-SH.)

As shown RT-PCR data in **Figure 9A**, the gene expression level of TNF-α in Group IV-VI is significantly lower than that of Group II, which might be attributed to the anti-inflammatory capability of DPCA@PDA and MMP-SP, which is confirmed by the data of TNF-α determined by immunostaining and RT-PCR. The similar trend with that of TNF-α is observed in the mRNA level of IL-1β in all the six groups (**Figure 9B**). Meanwhile, as exhibited in **Figure 9C**-**D**, the gene expression level of Ang-1 and HIF-1α in DCPA hydrogels (Group V and Group VI) is higher than that of other groups, which is due to the inhibition effect on PHDs. As indicated in **Figure 9E**, the expression of α-Actinin in the injured rat heart decreases sharply after MI, which declines to 20% level of normal heart, while the expression of α-Actinin is upregulated by the injection of the designed hydrogel. The ALG-CHO-TA/DPCA@PDA/MMP-SP/HA-SH hydrogel treated infracted rat hearts reveals the highest amount of α-Actinin to 80% level of normal heart. Both the soft hydrogel with biocompatible nature and the loading DPCA drug exerts their power on promote the expression of α-Actinin, which can be attributed to an enhance in cell to cell interactions and reversing the unfavorable microenvironment. As displayed in **Figure 9F**, the expression of cTnT demonstrates a similar tendency with that of α-Actinin.

After MI, myocardial ischemia leads to the death of a large number of myocardial cells and significant upregulation inflammatory factors, and then follows by a severe myocardial fibrosis deprived of the intercellular signal conduction 48, 55. With the rapid development of regenerative medicine and tissue engineering, a large number of hydrogels and cardiac patches have been developed to repair and reconstruct myocardial function. Reversing the hostile microenvironment of the wounded myocardium and promoting the formation of new blood vessels are the two most important aspects to improve the prognosis. Aiming to the above challenges, a multifunctional injectable hydrogel system based on natural biomacromolecules ALG and HA was reported in this report. The natural polymers with good biocompatibility were chosen to fabricate the injectable hydrogels, avoiding acute immune reactions to a large extent. To address the blocked electrical signal conduction in the myocardial infarction area, TA, which can be metabolized by the host due to a small molecular weight, was introduced into the hydrogel system to construct a conductive hydrogel. The conductive hydrogel can improve the signal conduction of myocardial cells in the myocardial infarction area as confirmed by the significant up-regulating of the expression of Cx43. At the same time, TA, PDA, and MMP-SP 42, 43, 56-58 in the hydrogel system can effectively slaughter ROS to reduce the expression level of inflammatory factors, witnessed by the down-regulating expression of TNF-α, IL-1β, and Cas-3. Moreover, the MMP-SP segments in the hydrogel can regulate the intelligent degradation of hydrogels, in turn achieving a controllable release of DPCA drug, and promoting cell migration and in-growth.

In this research, DPCA was self-assembled into nanodrug by hydrophobic interactions without any chemical modification. Then, DPCA nanodrugs were further modified and protected with PDA to greatly increase the drug loading amount and in order to achieve a stably controlled release profile. After being loaded in the hydrogels, the DPCA would be released in its intact chemical structure accompanying with the degradation of hydrogels. As a result, a favorable biosafety can be achieved by the local delivery of therapeutic drug. A large number of studies have shown that the myocardium of adult animals has a certain regeneration ability under the condition of stable expression of HIF-1α 33, 59, 60. As an inhibitor of PHDs, DPCA can efficiently stabilize HIF-1α to suppress the apoptosis of CMs and promote the formation of collateral circulation. In this study, a controllable release behavior of DCPA is obtained, resulting in an effective stabilization of HIF-1α for a long time, which can significantly increase the vascularization level of the infarcted area and promote tissue repair. All in all, the multifunctional hydrogel with good biocompatibility was synthesized to effectively improve the microenvironment of the infarcted area and rescue infarcted heart functions.

Apart from the promising outcomes presented in this report suggesting that ALG-CHO-TA/DPCA@PDA/MMP-SP/HA-SH hydrogel can be administrated through a minimally invasive intramyocardial injection and accomplish a powerful strength in promoting therapeutic effects on MI, three limitations remain and should be noted. Firstly, although a possible mechanism has been proposed as explained above, the accurate signaling pathways are unclear. Secondly, to verify the anti-inflammatory capacity, the as-prepared hydrogels were injected in the injured hearts immediately after MI, which would be different from a real clinical application of the injectable biomaterials. In the future research, an optimized timing of delivery and a more minimally invasive procedure through cardiac catheter to transport the designed injectable hydrogels into the infarcted area should be explored to fully meet the clinical applications. Thirdly, due to the immortalized cells being employed to evaluate hydrogel/cells interactions *in vitro*, the interactions between biomaterials and cardiac related cells (primary cardiomyocytes and fibroblasts) need to be explored in detail in future studies.

## Conclusion

To recover heart functions, HA/ALG hydrogel tethered by MMP sensitive peptides and loaded with DPCA@PDA nanodrug was elaborately designed to address the tough challenge of MI therapy. The conductivity of hydrogels enhances the expression of Cx43 by enhancing intercellular signal conduction. The controllable releasing properties of DPCA effectively improve heart functions and promote the regeneration of blood vessels by stabling the expression of HIF-1α, both of which contribute to the reversing of the ventricular remodeling and preserving heart functions after MI.

## Abbreviations

ALG: sodium alginate
ALG-CHO: oxidized alginate
ANG-1: Angiopoietin-1
cTnT: cardiac troponin
Cx43: connexin 43
DLS: dynamic light scattering
DMF: N, N-dimethylformamide
DOPA: dopamine hydrochloride
DPCA: 1,4-dihydrophenonthrolin-4-one-3-carboxylic acid
EF: ejection fraction
FS: fractional shortening
FTIR: fourier transform infrared
HA-SH: thiolated hyaluronic acid
HIF-1α: hypoxia-inducible factor-1α
^1^H NMR: proton nuclear magnetic resonance
ICG: indocyanine green
IL-1β: interleukin-1β
IVS: interventricular septum thickness
LV: left ventricular
LVID: left ventricular internal diameter
LVPW: left ventricular posterior wall thickness
MI: myocardial infarction
MMP-SP: matrix metalloproteinase-sensitive peptides
NaIO4: sodium periodate
NPs: nanoparticles
PDA: polymerized dopamine
PEG: polyethylene glycol
PHD: prolyl hydroxylase
ROS: reactive oxygen species
TA: tetraaniline
TEM: transmission electron microscope
TNF-α: tumor necrosis factor
UV-Vis: ultraviolet-visible spectrophotometry
WB: western blotting

## References

[1] Virani SS, Alonso A, Benjamin EJ, Bittencourt MS, Callaway CW, Carson AP (2020). Heart disease and stroke statistics-2020 update: a report from the American Heart Association. Circulation.

[2] Thavapalachandran S, Grieve SM, Hume RD, Le TYL, Raguram K, Hudson JE (2020). Platelet-derived growth factor-AB improves scar mechanics and vascularity after myocardial infarction. Sci Transl Med.

[3] Langin M, Mayr T, Reichart B, Michel S, Buchholz S, Guethoff S (2018). Consistent success in life-supporting porcine cardiac xenotransplantation. Nature.

[4] Hasan A, Khattab A, Islam MA, Hweij KA, Zeitouny J, Waters R (2015). Injectable hydrogels for cardiac tissue repair after myocardial Infarction. Adv Sci.

[5] Wang LL, Liu Y, Chung JJ, Wang T, Gaffey AC, Lu MM (2017). Sustained miRNA delivery from an injectable hydrogel promotes cardiomyocyte proliferation and functional regeneration after ischaemic injury. Nat Biomed Eng.

[6] Leor J, Tuvia S, Guetta V, Manczur F, Castel D, Willenz U (2009). Intracoronary injection of in situ forming alginate hydrogel reverses left ventricular remodeling after myocardial infarction in swine. J Am Coll Cardiol.

[7] Sabbah HN, Wang MJ, Gupta RC, Rastogi S, Ilsar I, Sabbah MS (2013). Augmentation of left ventricular wall thickness with alginate hydrogel implants improves left ventricular function and prevents progressive remodeling in dogs with chronic heart failure. Jacc-Heart Fail.

[8] Rao SV, Zeymer U, Douglas PS, Al-Khalidi H, White JA, Liu JY (2016). Bioabsorbable intracoronary matrix for prevention of ventricular remodeling after myocardial infarction. J Am Coll of Cardiol.

[9] He HJ, Zhao YN, Zhang H, Wang B, Pan J, Li J (2020). Effect of intramyocardial grafting collagen scaffold with mesenchymal stromal cells in patients with chronic ischemic heart disease a randomized clinical trial. JAMA Netw Open.

[10] Schirmer L, Chwalek K, Tsurkan MV, Freudenberg U, Werner C (2020). Glycosaminoglycan-based hydrogels with programmable host reactions. Biomaterials.

[11] Zhu Y, Jiang H, Ye SH, Yoshizumi T, Wagner WR (2015). Tailoring the degradation rates of thermally responsive hydrogels designed for soft tissue injection by varying the autocatalytic potential. Biomaterials.

[12] Rane AA, Chuang JS, Shah A, Hu DP, Dalton ND, Gu YS (2011). Increased infarct wall thickness by a bio-inert material is insufficient to prevent negative left ventricular remodeling after myocardial infarction. Plos One.

[13] Lutolf MP, Lauer-Fields JL, Schmoekel HG, Metters AT, Weber FE, Fields GB (2003). Synthetic matrix metalloproteinase-sensitive hydrogels for the conduction of tissue regeneration: Engineering cell-invasion characteristics. P Natl Acad Sci USA.

[14] Purcell BP, Lobb D, Charati MB, Dorsey SM, Wade RJ, Zellars KN (2014). Injectable and bioresponsive hydrogels for on-demand matrix metalloproteinase inhibition. Nat Mater.

[15] Deleon-Pennell KY, Altara R, Yabluchanskiy A, Modesti A, Lindsey ML (2015). The circular relationship between matrix metalloproteinase-9 and inflammation following myocardial infarction. Iubmb Life.

[16] Fan CX, Shi JJ, Zhuang Y, Zhang LL, Huang L, Yang W (2019). Myocardial-infarction-responsive smart hydrogels targeting matrix metalloproteinase for on-demand growth factor delivery. Adv Mater.

[17] Ban K, Park HJ, Kim S, Andukuri A, Cho KW, Hwang JW (2014). Cell therapy with embryonic stem cell-derived cardiomyocytes encapsulated in injectable nanomatrix gel enhances cell engraftment and promotes cardiac repair. ACS Nano.

[18] Lee SJ, Sohn YD, Andukuri A, Kim S, Byun J, Han JW (2017). Enhanced therapeutic and long-term dynamic vascularization effects of human pluripotent stem cell-derived endothelial cells encapsulated in a nanomatrix gel. Circulation.

[19] Mihic A, Cui Z, Wu J, Vlacic G, Miyagi Y, Li SH (2015). A conductive polymer hydrogel supports cell electrical signaling and improves cardiac function after implantation into myocardial infarct. Circulation.

[20] Dong R, Ma PX, Guo BL (2020). Conductive biomaterials for muscle tissue engineering. Biomaterials.

[21] Zhang CY, Hsieh MH, Wu SY, Li SH, Wu J, Liu SM (2020). A self-doping conductive polymer hydrogel that can restore electrical impulse propagation at myocardial infarct to prevent cardiac arrhythmia and preserve ventricular function. Biomaterials.

[22] Burnstine-Townley A, Eshel Y, Amdursky N (2020). Conductive scaffolds for cardiac and neuronal tissue engineering: governing factors and mechanisms. Adv Funct Mater.

[23] Walker BW, Lara RP, Mogadam E, Yu CH, Kimball W, Annabi N (2019). Rational design of microfabricated electroconductive hydrogels for biomedical applications. Prog Polym Sci.

[24] Wu YB, Wang L, Guo BL, Ma PX (2017). Interwoven aligned conductive nanofiber yarn/hydrogel composite scaffolds for engineered 3D cardiac anisotropy. ACS Nano.

[25] Liang W, Chen JR, Li LY, Li M, Wei XJ, Tan BY (2019). Conductive hydrogen sulfide-releasing hydrogel encapsulating ADSCs for myocardial infarction treatment. ACS Appl Mater Inter.

[26] Wang W, Tan BY, Chen JR, Bao R, Zhang XR, Liang S (2018). An injectable conductive hydrogel encapsulating plasmid DNA-eNOs and ADSCs for treating myocardial infarction. Biomaterials.

[27] Thangarajah H, Yao D, Chang EI, Shi Y, Jazayeri L, Vial IN (2009). The molecular basis for impaired hypoxia-induced VEGF expression in diabetic tissues. P Natl Acad Sci USA.

[28] Eltzschig HK, Bratton DL, Colgan SP (2014). Targeting hypoxia signalling for the treatment of ischaemic and inflammatory diseases. Nat Rev Drug Discov.

[29] Zhang Y, Strehin I, Bedelbaeva K, Gourevitch D, Clark L, Leferovich J (2015). Drug-induced regeneration in adult mice. Sci Transl Med.

[30] Stoehr A, Yang YQ, Patel S, Evangelista AM, Aponte A, Wang GH (2016). Prolyl hydroxylation regulates protein degradation, synthesis, and splicing in human induced pluripotent stem cell-derived cardiomyocytes. Cardiovasc Res.

[31] Heber-Katz E, Messersmith P (2018). Drug delivery and epimorphic salamander-type mouse regeneration: A full parts and labor plan. Adv Drug Deliver Rev.

[32] Esser TU, Roshanbinfar K, Engel FB (2019). Promoting vascularization for tissue engineering constructs: current strategies focusing on HIF-regulating scaffolds. Expert Opin Biol Ther.

[33] Cheng J, Amin D, Latona J, Heber-Katz E, Messersmith PB (2019). Supramolecular polymer hydrogels for drug-induced tissue regeneration. ACS Nano.

[34] Nagai K, Ideguchi H, Kajikawa T, Li XF, Chavakis T, Cheng J (2020). An injectable hydrogel-formulated inhibitor of prolyl-4-hydroxylase promotes T regulatory cell recruitment and enhances alveolar bone regeneration during resolution of experimental periodontitis. Faseb J.

[35] Tambuwala MM, Manresa MC, Cummins EP, Aversa V, Coulter IS, Taylor CT (2015). Targeted delivery of the hydroxylase inhibitor DMOG provides enhanced efficacy with reduced systemic exposure in a murine model of colitis. J Control Release.

[36] Piantanida E, Alonci G, Bertucci A, De Cola L (2019). Design of Nanocomposite injectable hydrogels for minimally invasive surgery. Acc Chem Res.

[37] Park J, Brust TF, Lee HJ, Lee SC, Watts VJ, Yeo Y (2014). Polydopamine-based simple and versatile surface modification of polymeric nano drug carriers. ACS Nano.

[38] Cheng W, Nie JP, Gao NS, Liu G, Tao W, Xiao XJ (2017). A multifunctional nanoplatform against multidrug resistant cancer: merging the best of targeted Chemo/Gene/Photothermal therapy. Adv Funct Mater.

[39] Wu YH, Wang HB, Gao F, Xu ZY, Dai FY, Liu WG (2018). An injectable supramolecular polymer nanocomposite hydrogel for prevention of breast cancer recurrence with theranostic and mammoplastic functions. Adv Funct Mater.

[40] Wang W, Chen JR, Li M, Jia HZ, Han XX, Zhang JX (2019). Rebuilding postinfarcted cardiac functions by injecting TIIA@PDA nanoparticle-cross-linked ROS-sensitive hydrogels. ACS Appl Mater Inter.

[41] Han X, Li L, Xie T, Chen S (2020). “Ferrero-like” nanoparticles knotted injectable hydrogels to initially scavenge ROS and lastingly promote vascularization in infarcted hearts. Sci China Tech Sci.

[42] Bao XF, Zhao JH, Sun J, Hu M, Yang XR (2018). Polydopamine Nanoparticles as Efficient Scavengers for Reactive Oxygen Species in Periodontal Disease. ACS Nano.

[43] Wang X, Wang CP, Wang XY, Wang YT, Zhang Q, Cheng YY (2017). A Polydopamine nanoparticle-knotted poly(ethylene glycol) hydrogel for on-demand drug delivery and chemo-photothermal therapy. Chem Mater.

[44] Zhu Y, Matsumura Y, Wagner WR (2017). Ventricular wall biomaterial injection therapy after myocardial infarction: Advances in material design, mechanistic insight and early clinical experiences. Biomaterials.

[45] Ruvinov E, Cohen S (2016). Alginate biomaterial for the treatment of myocardial infarction: Progress, translational strategies, and clinical outlook From ocean algae to patient bedside. Adv Drug Deliver Rev.

[46] Balakrishnan B, Joshi N, Jayakrishnan A, Banerjee R (2014). Self-crosslinked oxidized alginate/gelatin hydrogel as injectable, adhesive biomimetic scaffolds for cartilage regeneration. Acta Biomater.

[47] Hua YJ, Gan YB, Zhang YQ, Ouyang B, Tu B, Zhang CM (2019). Adaptable to mechanically stable hydrogels Based on the dynamic covalent cross-linking of thiol-aldehyde addition. ACS Macro Lett.

[48] Liang S, Zhang Y, Wang H, Xu Z, Chen J, Bao R (2018). Paintable and rapidly bondable bonductive hydrogels as therapeutic cardiac patches. Adv Mater.

[49] Qazi TH, Rai R, Dippold D, Roether JE, Schubert DW, Rosellini E (2014). Development and characterization of novel electrically conductive PANI-PGS composites for cardiac tissue engineering applications. Acta Biomater.

[50] Broughton KM, Wang BJ, Firouzi F, Khalafalla F, Dimmeler S, Fernandez-Aviles F (2018). Mechanisms of cardiac repair and regeneration. Circ Res.

[51] Yao Y, Ding J, Wang Z, Zhang H, Xie J, Wang Y (2020). ROS-responsive polyurethane fibrous patches loaded with methylprednisolone (MP) for restoring structures and functions of infarcted myocardium in vivo. Biomaterials.

[52] Yuan Z, Tsou YH, Zhang XQ, Huang S, Yang Y, Gao M (2019). Injectable citrate-based hydrogel as an angiogenic biomaterial improves cardiac repair after myocardial infarction. ACS Appl Mater Interfaces.

[53] Yang H, Qin X, Wang H, Zhao X, Liu Y, Wo HT (2019). An in vivo miRNA delivery system for restoring infarcted myocardium. ACS Nano.

[54] Dobaczewski M, Gonzalez-Quesada C, Frangogiannis NG (2010). The extracellular matrix as a modulator of the inflammatory and reparative response following myocardial infarction. J Mol Cell Cardiol.

[55] Feiner R, Engel L, Fleischer S, Malki M, Gal I, Shapira A (2016). Engineered hybrid cardiac patches with multifunctional electronics for online monitoring and regulation of tissue function. Nat Mater.

[56] Guo BL, Ma PX (2018). Conducting polymers for tissue engineering. Biomacromolecules.

[57] Cui H, Liu Y, Cheng Y, Zhang Z, Zhang P, Chen X (2014). In vitro study of electroactive tetraaniline-containing thermosensitive hydrogels for cardiac tissue engineering. Biomacromolecules.

[58] Zhou T, Yan LW, Xie CM, Li PF, Jiang LL, Fang J (2019). A Mussel-inspired persistent ROS-scavenging, electroactive, and osteoinductive scaffold based on electrochemical-driven in Situ nanoassembly. Small.

[59] Nakada Y, Canseco DC, Thet S, Abdisalaam S, Asaithamby A, Santos CX (2017). Hypoxia induces heart regeneration in adult mice. Nature.

[60] Kimura W, Xiao F, Canseco DC, Muralidhar S, Thet S, Zhang HM (2015). Hypoxia fate mapping identifies cycling cardiomyocytes in the adult heart. Nature.

