# Supplementary material for: An MMP-degradable and conductive hydrogel to stabilize HIF-1α for recovering cardiac functions
Source: Theranostics. 2022 Jan 1;12(1):127–42. doi: 10.7150/thno.63481 (PMC8690911; doi:10.7150/thno.63481)
Supplement: Supplementary file 1 — Supplementary figures and tables. [file thnov12p0127s1.pdf]

SUPPLEMENTARY DATA

An MMP-degradable and conductive hydrogel to stabilize HIF-1α for recovering cardiac functions

Xiaojuan Wei [1 #], Si Chen [2,3,4 #], Tian Xie [3], Hongchi Chen [5], Xin Jin [3], Jumin Yang [3], Shafaq Sahar [2], Huanlei Huang [6,7], Shuoji Zhu [6,7], Nanbo Liu [6,7], Changjiang Yu [6,7], Ping Zhu [6,7] *, Wei Wang[2,3,4] *, Wei Zhang [1,5] *

1 Institute of Microsurgery on Extremities, Shanghai Jiao Tong University Affiliated Shanghai Sixth People's Hospital, Shanghai 200233, China.

2 College of Chemical and Biological Engineering, Zhejiang University, Hangzhou 310027, China.

3 School of Materials Science and Engineering, Tianjin Key Laboratory of Composite and Functional Materials, Tianjin University, Tianjin 300350, China.

4. ZJU-Hangzhou Global Scientific and Technological Innovation Center, Hangzhou 311215, China.

5. Department of Orthopedic Surgery, Shanghai Jiao Tong University Affiliated Shanghai Sixth People's Hospital, Shanghai 200233, China.

6 Guangdong Cardiovascular Institute, Guangdong Provincial People's Hospital, Guangdong Academy of Medical Sciences, Guangzhou, Guangdong 510100, China.

7 Department of Cardiovascular Surgery, Sun Yat-sen Memorial Hospital, Sun Yat-sen University, Guangzhou, Guangdong 510100, China.

# These authors contributed equally to this paper.

E-mail address: Prof. Ping Zhu, tanganqier@163.com; Prof. Wei Wang, wwgfz@zju.edu.cn;

Prof. Wei Zhang, orthozhang@163.com.

Table S1 the components and gelation time of the as-prepared hydrogels

Group	ALG-CHO (wt%)	ALG-CHO-TA (wt%)	DPCA@PDA (wt%)	MMP-SP (wt%)	HA-SH (wt%)	Gelation time (s)
ALG-CHO/HA-SH (0.67%)	0.2	0	0	0	0.67	83.3±6.5
ALG-CHO/HA-SH (1.33%)	0	0.2	0	0	1.33	27.3±2.1
ALG-CHO-TA/HA-SH	0	0.2	0	0	1.33	24.3±5.0
ALG-CHO-TA/ DPCA@PDA/HA-SH	0	0.2	0.01	0	1.33	23.7±3.1
ALG-CHO-TA/ DPCA@PDA/ MMP-SP/HA-SH	0	0.2	0.01	0.02	1.33	25.0±4.4

Table S2 the conductivity of hydrogels after being immersed in PBS

Time (h)	Conductivity (S/cm)
0	$8.9 \pm 0.3 \times 10^{-5}$
3	$9.5 \pm 0.8 \times 10^{-5}$
8	$8.7 \pm 1.1 \times 10^{-5}$
24	$9.9 \pm 1.3 \times 10^{-5}$

Table S3 the antibody used in this research

Antibody	Company	Clone number
TNF-α	Abcam	ab6671
HIF-α	Abcam	ab2185
VEGFA	Abcam	ab1316
α-SMA	Abcam	ab32575
cTnT	Abcam	ab8295
Tunel	Roche	11684817910
Cx43	Abcam	Ab11370
Caspase 3	CST	#9664

Table S4 RT-PCR primer sequence

Gene	Forward primer	Reverse primer
**TNF-α**	CGTGTTCATCCGTTCTCTACC	CTACTTCAGCGTCTCGTGTG
**IL-1β**	CTTGACTTGGGCTGTCCAGA	ACGGGCAAGACATAGGTAGC
**HIF-1α**	CAACTGCCACCACTGATGAATC	ACCACTGTATGCTGATGCCTTAG
**Ang-1**	AGGAAACCAGAAGCAGAACTACAG	ACAGGCATCAAACCACCAACC
**α-Actinin**	CCTTCAACAACTGGATGGAG	TGGACAATCTTGGACACTTC
**cTnT**	AGGAGGAAGGCTGAAGATGAG	TTCTCTCGCTCTGTCTGTCTC

Table S5 the survival rate of rats in each group within 28 days

Group	0 d	3 d	7 d	14 d	28 d	Survival rate
I	12	12-2-0	10-2-0	8-2-0	6-6-0	100%
II	21	21-3-3	15-3-0	12-3-1	8-6-2	71.4%
III	21	21-4-2	15-4-0	11-4-1	6-5-1	81.0%
IV	21	21-4-2	15-4-0	11-4-1	6-6-0	85.7%
V	21	21-4-2	15-4-1	10-4-0	6-6-0	85.7%
VI	21	21-4-2	15-4-0	11-4-0	7-7-0	90.5%

The black numbers (start number) represent the number of surviving rats at that time, the red numbers (end number) represent the number of rats killed at this time point for testing, while the blue numbers (mid number) represent natural death from the last time to this time point. None rat died in the Sham group (Group I), while three rats died the MI group (Group II) in the early stage (the first 3 d) and three in the later stage (7-28 d), while the mortality rate of the four groups in the experimental group was reduced in the later period. The MI group (Group II) was injected with 0.9% NaCl after MI model.

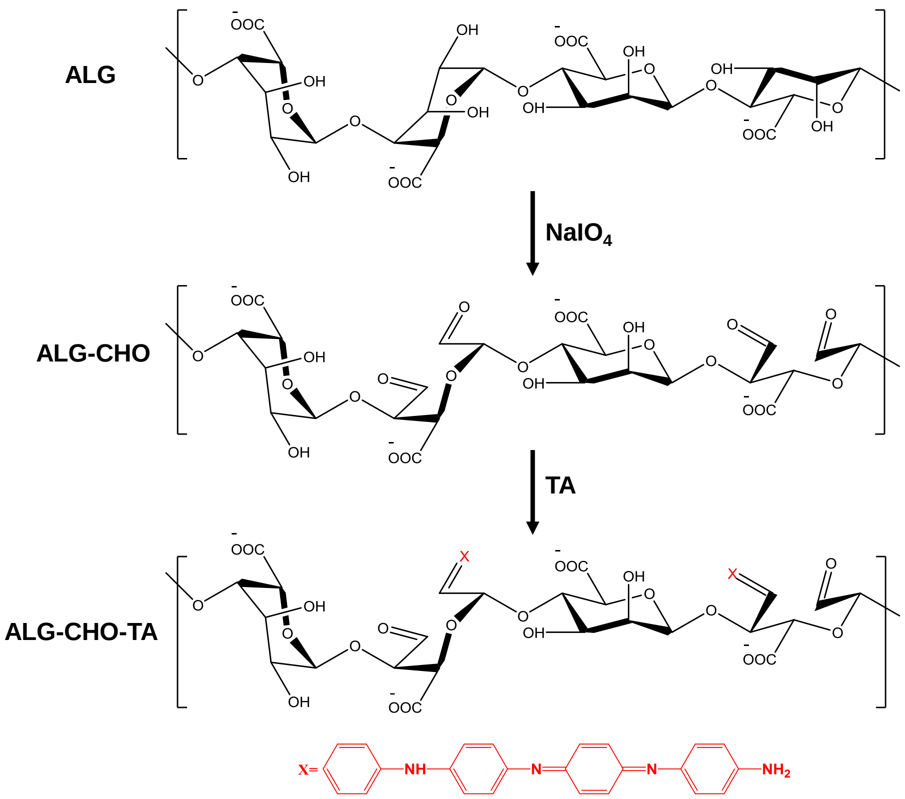

Figure S1 Chemical synthesis of ALG-CHO-TA

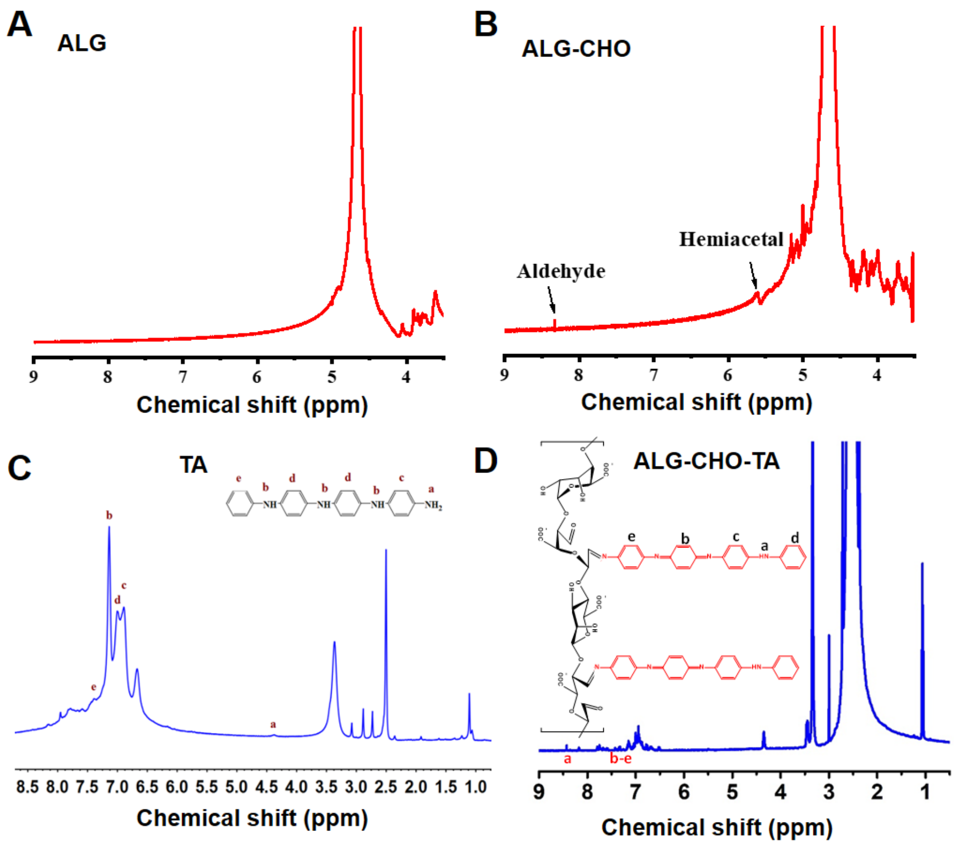

Figure S2 ^{1}H-NMR spectra of (A) ALG, (B) ALG-CHO, (C) TA, and (D) ALG-CHO-TA

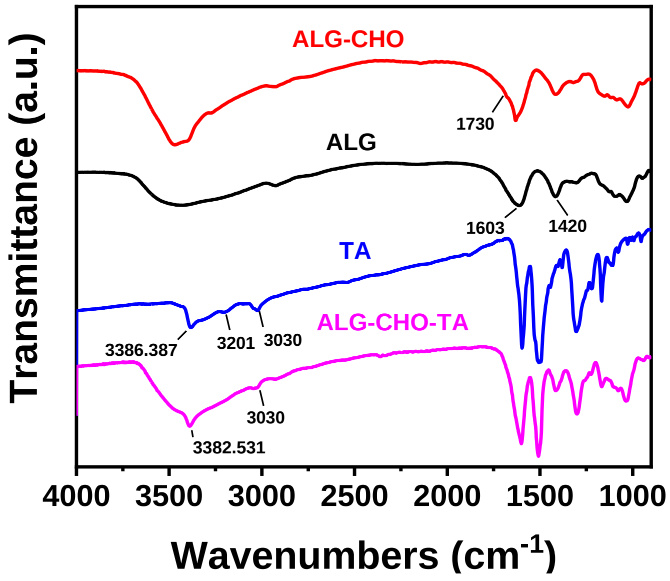

Figure S3 FTIR spectra of TA, ALG, ALG-CHO, and ALG-CHO-TA

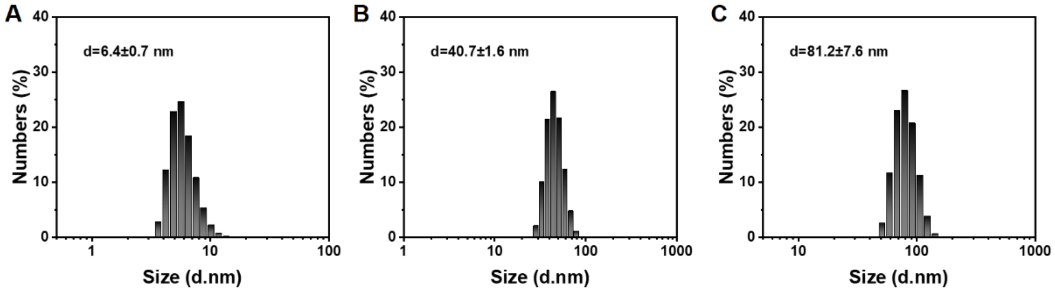

Figure S4 Hydrodynamic size of DPCA NPs with various DPCA concentrations. The concentrations of DPCA of 1 (A), 2 (B), and 5 mg/mL (C), respectively.

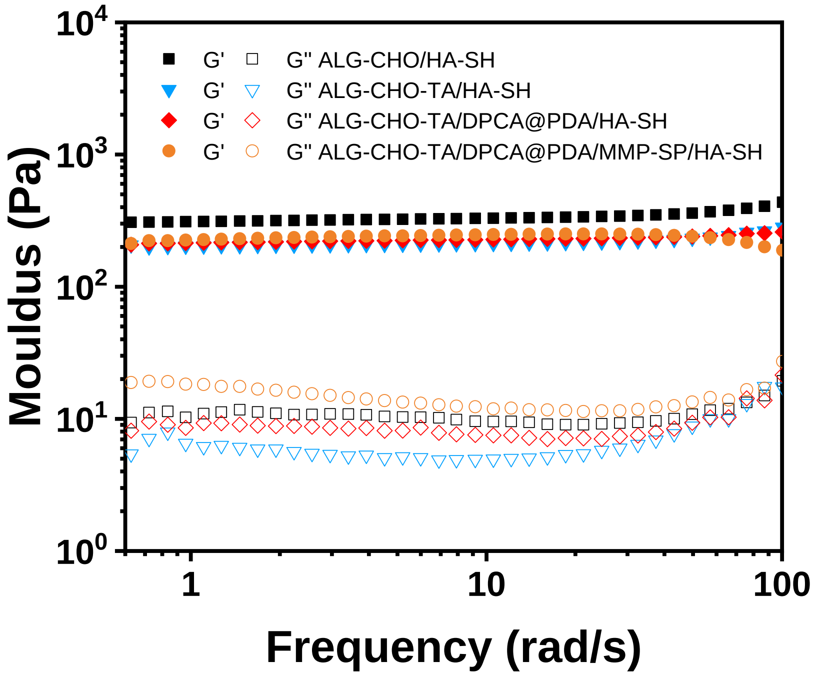

Figure S5 The frequency-sweep of the synthesized hydrogels

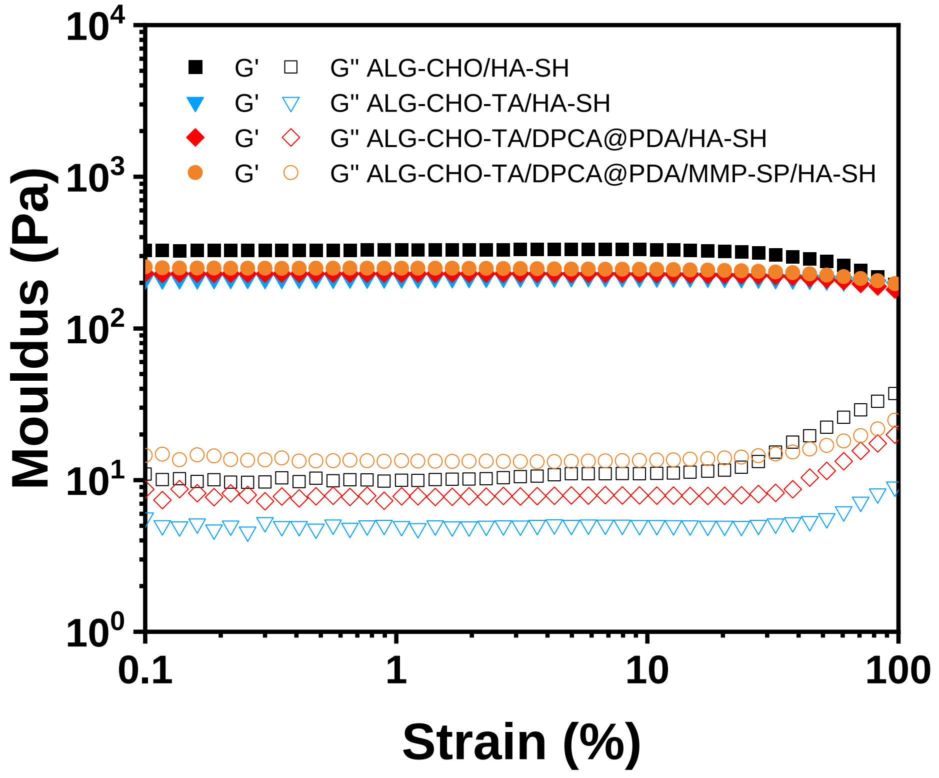

Figure S6 The strain-sweep of the synthesized hydrogels

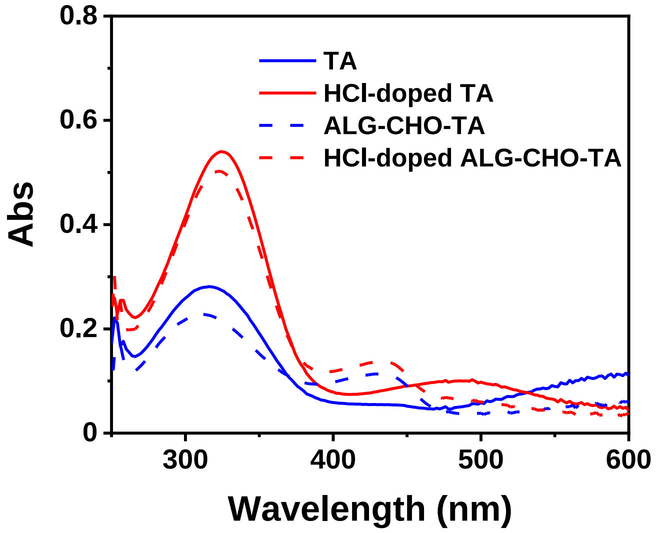

Figure S7 UV-vis spectra of TA and ALG-CHO-TA

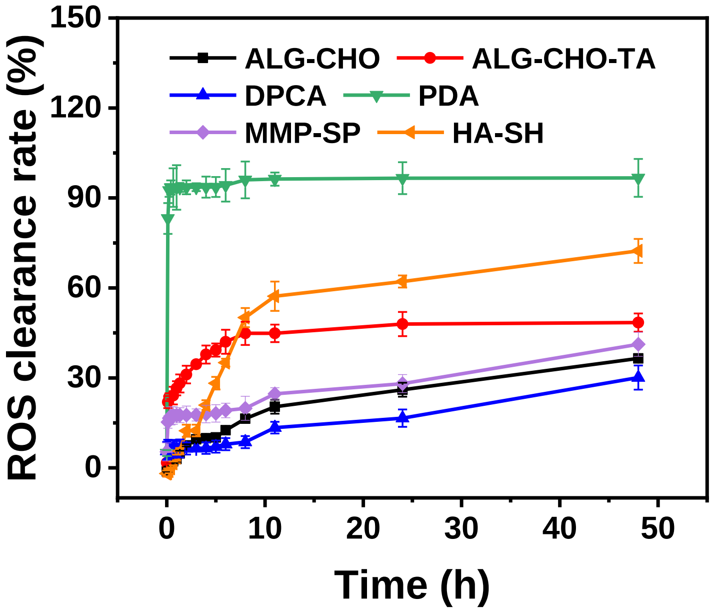

Figure S8 ROS clearance rates of the hydrogel's components *in vitro*

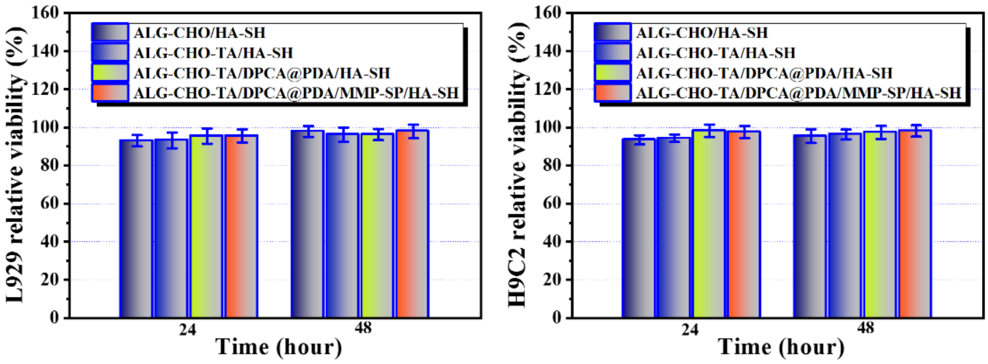

Figure S9 Relative CMs viability of the hydrogels assayed by a typical MTT method.

H9C2 or L929 cells were seeded in a 96-well plate at 1×10^4 cells per well and incubated for 24 hours at 37 ºC in 5 % CO_2 humidified atmosphere. Then the culture medium was removed and 100 μL hydrogel extract and 100 μL cell culture medium were mixed and added to each well. After incubation for 24 h and 48 h, the culture medium was removed and MTT kit was used to test cell viability according to the manufacturer's instructions. The relative viability of the cells was calculated with a control in which the cell viability in the cell culture medium was set as 100%.

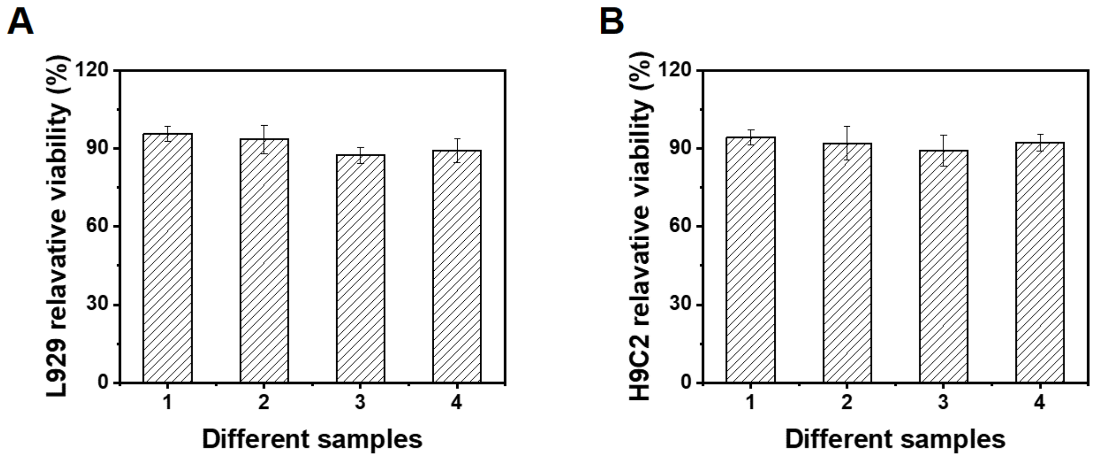

Figure S10 The cytocompatibility characterized by cells (A, L929 cells; B, H9C2 cells) on the top of hydrogels after 24 hours. The hydrogel samples are (1) ALG-CHO/HA-SH, (2) ALG-CHO-TA/HA-SH, (3) ALG-CHO-TA/DPCA@PDA/HA-SH, and (4) ALG-CHO-TA/DPCA@PDA/MMP-SP/HA-SH.

The cell viability was characterized as the method shown in Figure S9.

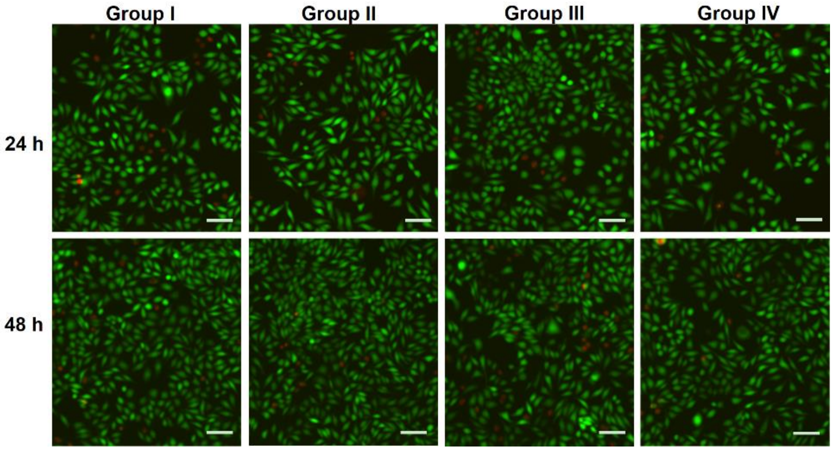

Figure S11 The live/dead viability of H9C2. The scale bar is 50 μm. Group I-IV indicates (I) ALG-CHO/HA-SH, (II) ALG-CHO-TA/HA-SH, (III) ALG-CHO-TA/DPCA@PDA/HA-SH, and (IV) ALG-CHO-TA/DPCA@PDA/MMP-SP/HA-SH.

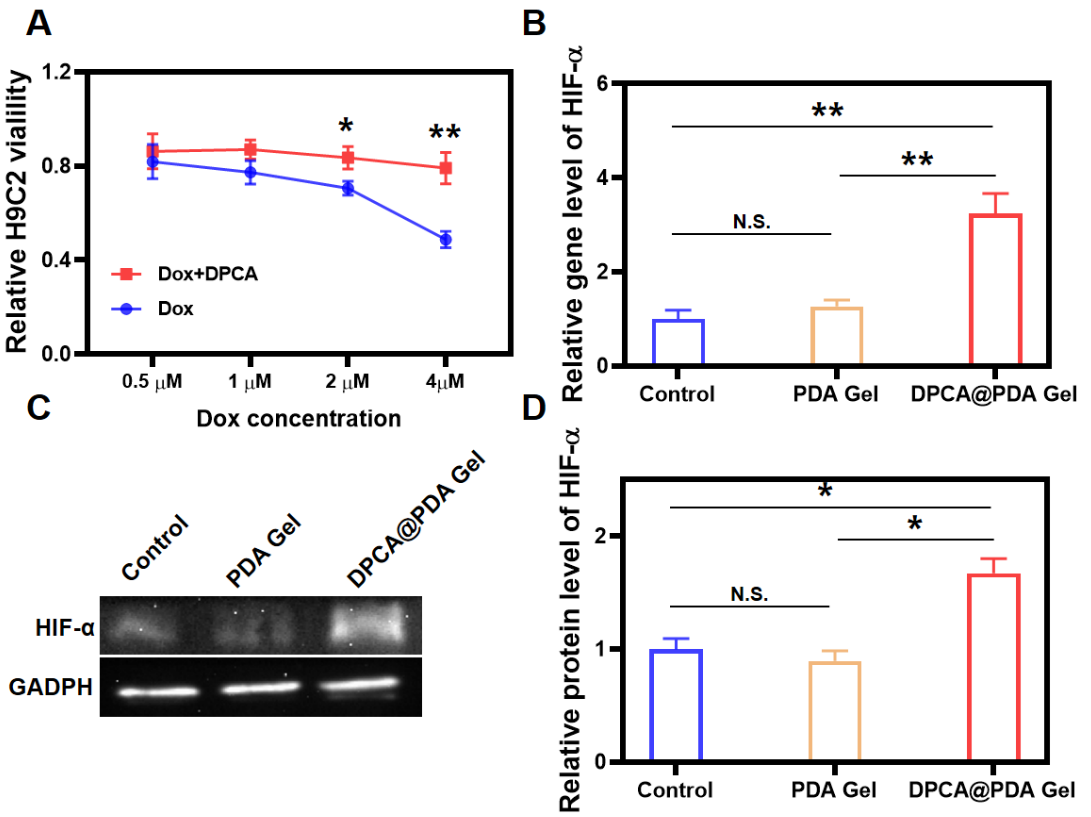

Figure S12 (A) Protective effect of DCPA from Dox for H9C2; (B) HIF-1α expression of H9C2 co-cultured with hydrogels by PCR; (C) HIF-1α expression of H9C2 co-cultured with hydrogels by WB; (D) Quantitative results of WB by Image J software. Results were shown as the average values ± s.d. (n = 3).

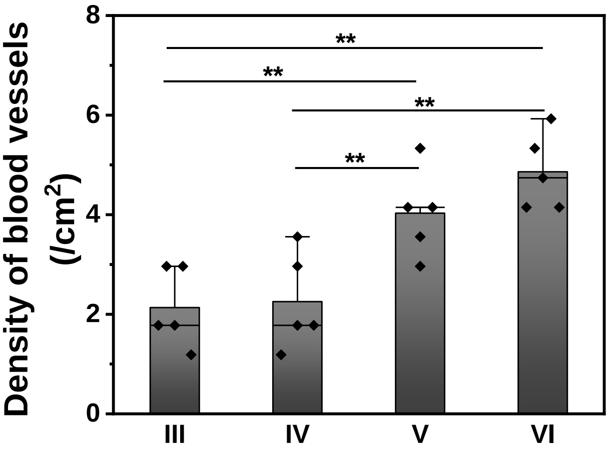

Figure S13 The vascular densities around hydrogels. (III: ALG-CHO/HA-SH; IV: ALG-CHO-TA/HA-SH; V: ALG-CHO-TA/DPCA@PDA/HA-SH; VI: ALG-CHO-TA/DPCA@PDA/MMP-SP/HA-SH.)

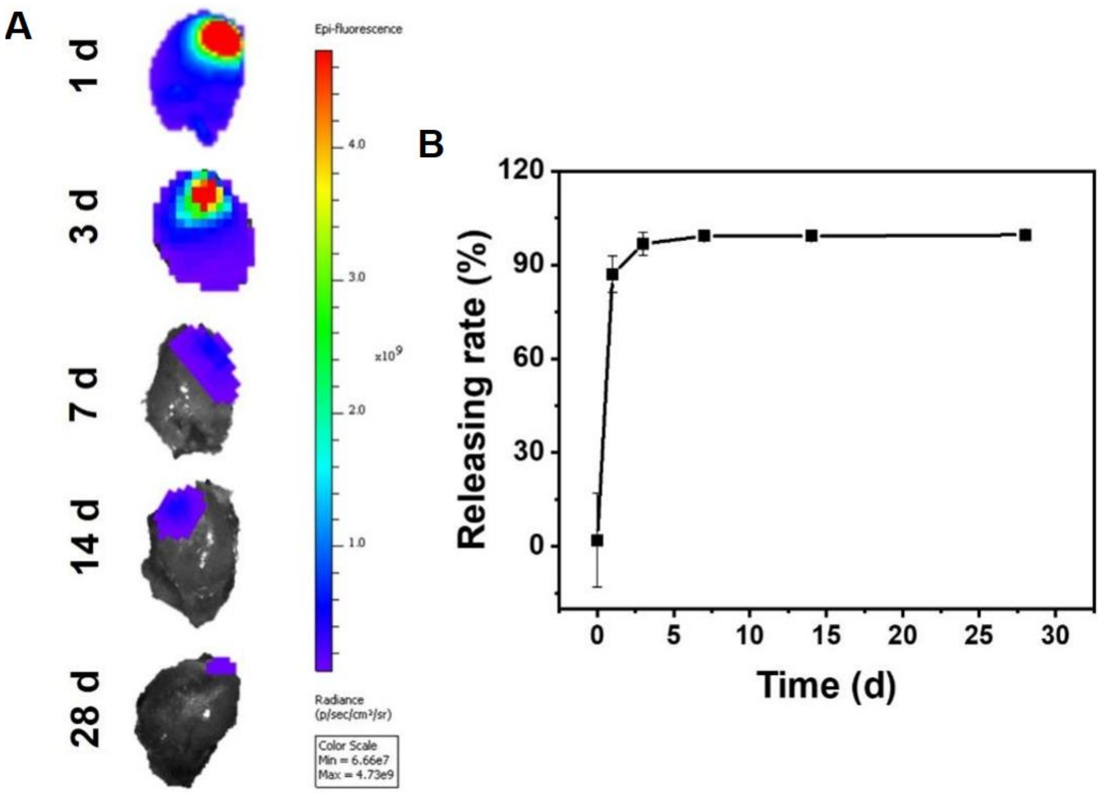

Figure S14 Drug releasing behavior *in vivo*. (A) Fluorescence picture of ICG in the heart. (B) The releasing rate of ICG *in vivo*.

Indocyanine green (ICG) is one of the FDA approved photosensitizers, which is safe and widely used in bioimaging and diagnose. In order to determine the drug releasing behavior in vivo, the fluorescent drug ICG instead of DPCA has been employed to prepare nanoparticles with the same procedure, and the resulted ICG nanoparticles was coated with PDA and crosslinked with the ALG-CHO-TA/MMP-SP/HA-SH hydrogels that were injected into the rat hearts to test the drug releasing behavior *in vivo*. As shown in Figure S14, the fluorescent intensity of ICG in the heart is rapidly weakened within the first three days. As calculated by the Image J, the drug releasing rate exceeds 95% after 3 days. In addition, subcutaneous injection experiments show that the hydrogel with MMP-SP has a faster degradation rate, indicating that the gel possesses the controllable drug releasing behavior in response.

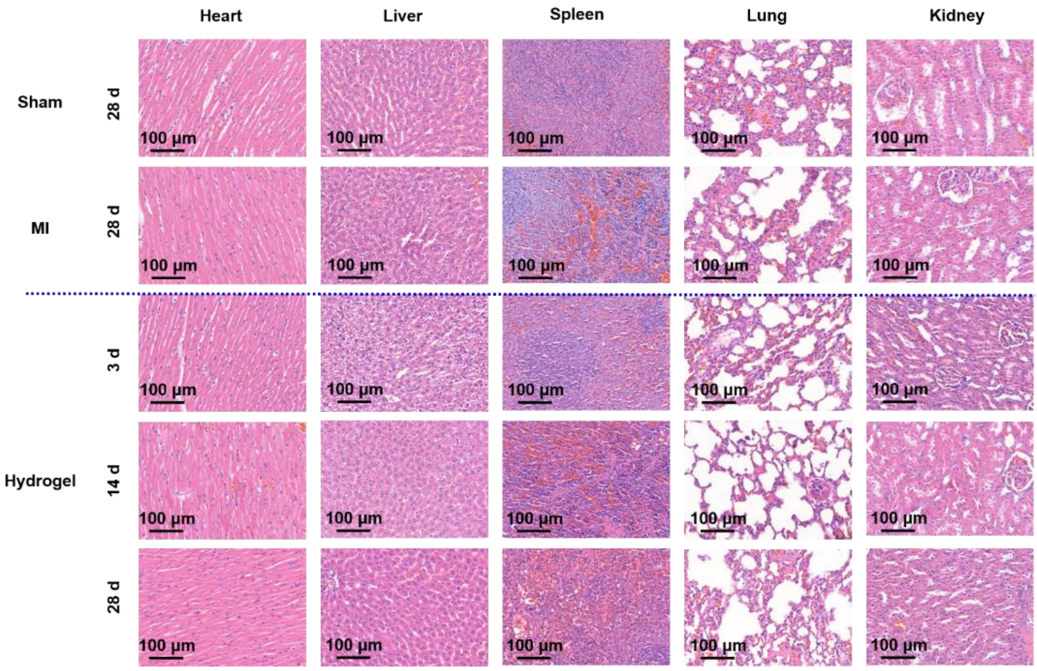

Figure S15 HE staining of the main organs (heart, liver, spleen, lung, and kidney) of rats after being treated by ALG-CHO-TA/DPCA@PDA/MMP-SP/HA-SH hydrogels.

As indicated in **Figure S15**, the main organs (heart, liver, spleen, lung, and kidney) show no serious lesions or abnormalities and significant inflammatory response. The experimental results reveal that the injection of hydrogels with TA NPs and DCPA NPs would not cause severe inflammation and other obvious negative effects on the main organs of rats.

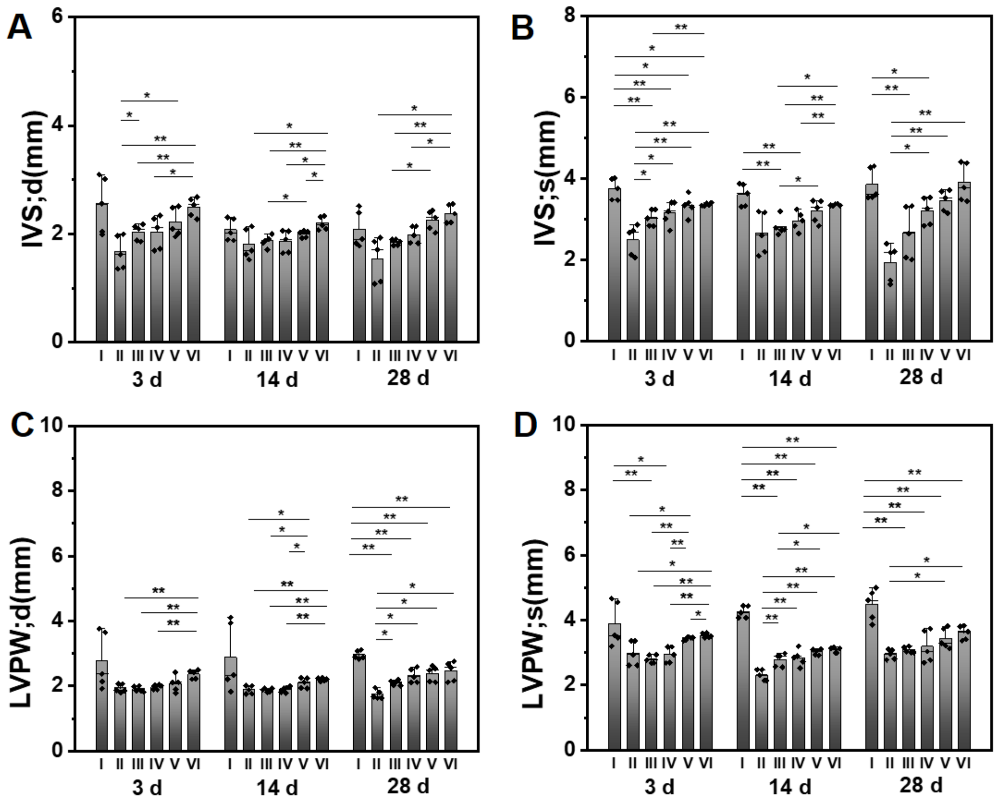

Figure S16 The statistical data of (A) IVS; d, (B) IVS; s, (C) LVPW; d, and (D) LVPW; s. (I: Sham; II: MI; III: ALG-CHO/HA-SH; IV: ALG-CHO-TA/HA-SH; V: ALG-CHO-TA/DPCA@PDA/HA-SH; VI: ALG-CHO-TA/DPCA@PDA/MMP-SP/HA-SH.)

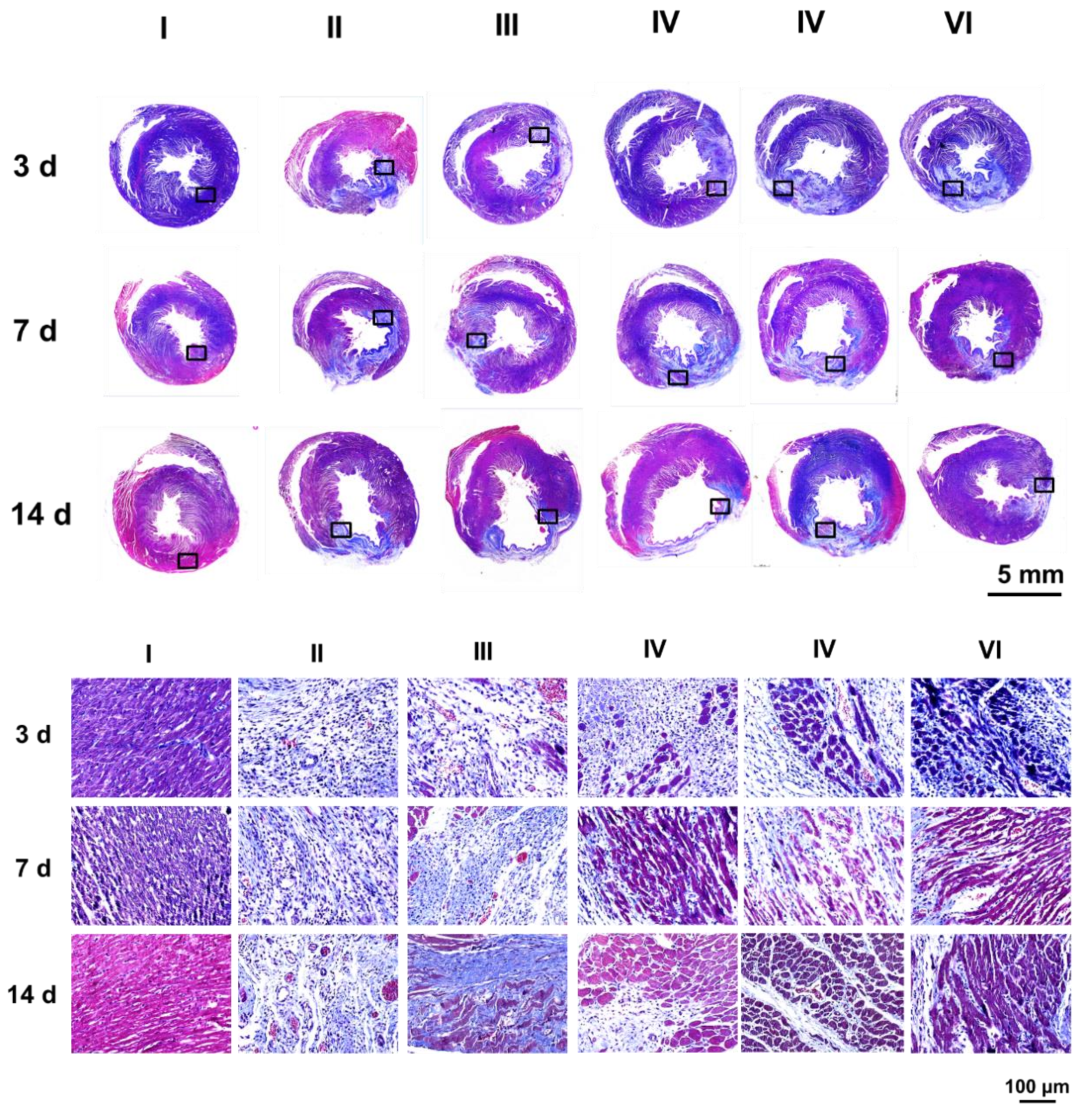

Figure S17 Masson staining of the rat hearts after MI at various time period (I: Sham; II: MI; III: ALG-CHO/HA-SH; IV: ALG-CHO-TA/HA-SH; V: ALG-CHO-TA/DPCA@PDA/HA-SH; VI: ALG-CHO-TA/DPCA@PDA/MMP-SP/HA-SH.)

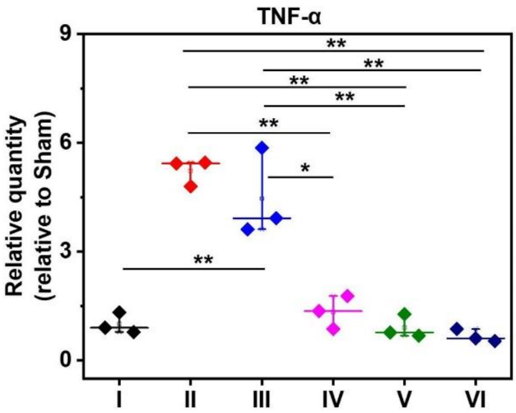

Figure S18 The expression of TNF-α after 28 days determined by the immunostaining method and normalized to the number of nuclei.

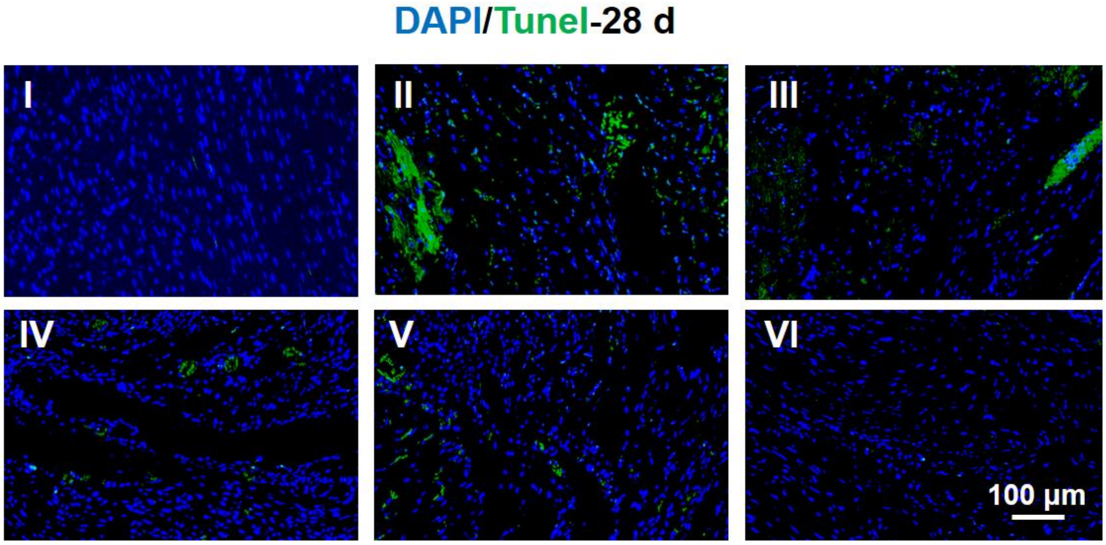

Figure S19 Tunel immunofluorescent staining at day 28 (I: Sham; II: MI; III: ALG-CHO/HA-SH; IV: ALG-CHO-TA/HA-SH; V: ALG-CHO-TA/DPCA@PDA/HA-SH; VI: ALG-CHO-TA/DPCA@PDA/MMP-SP/HA-SH.)

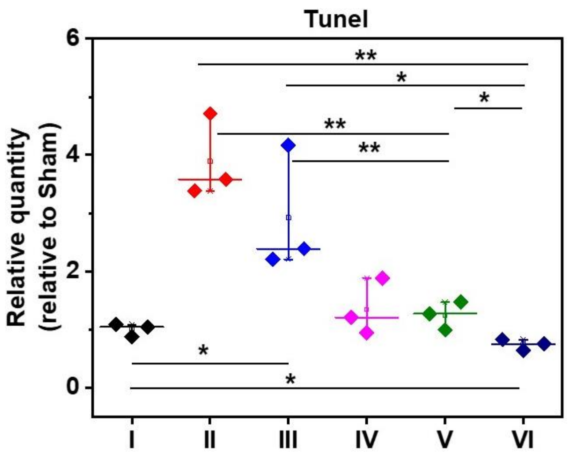

Figure S20 The expression of terminal deoxynucleotidyl transferase (TdT) at day 28 determined by a Tunel immunostaining method normalized to the number of nuclei.
